# The association between individual and neighbourhood-level measures of socioeconomic disadvantage and severe maternal morbidity, in the Netherlands, a retrospective cohort study

**DOI:** 10.1093/eurpub/ckag050

**Published:** 2026-04-07

**Authors:** Dorothea Geddes-Barton, Anouk Klootwijk, Rema Ramakrishnan, Raph Goldacre, Jessica C Kiefte-de Jong, Marian Knight

**Affiliations:** National Perinatal Epidemiology Unit, Nuffield Department of Population Health, University of Oxford, Oxford, United Kingdom; Faculty of Health and Life Sciences, St Luke’s Campus, University of Exeter, Exeter, United Kingdom; Department of Public Health and Primary Care/Health Campus The Hague, Leiden University Medical Center, Leiden, The Netherlands; Center for Public Health, Healthcare and Society, National Institute for Public Health and the Environment, Bilthoven, The Netherlands; National Perinatal Epidemiology Unit, Nuffield Department of Population Health, University of Oxford, Oxford, United Kingdom; Oxford NIHR Biomedical Research Centre, University of Oxford, Oxford, United Kingdom; Applied Health Research Unit, Nuffield Department of Population Health, University of Oxford, Oxford, United Kingdom; Department of Public Health and Primary Care/Health Campus The Hague, Leiden University Medical Center, Leiden, The Netherlands; National Perinatal Epidemiology Unit, Nuffield Department of Population Health, University of Oxford, Oxford, United Kingdom

## Abstract

Socioeconomic disadvantage is associated with severe maternal morbidity (SMM), across high-income countries. However, neighbourhood-level measures of disadvantage, often used in population-based studies, may underestimate the effect of individual socioeconomic disadvantage. This study aimed to compare the strength of the associations between individual- and neighbourhood-level measures of socioeconomic disadvantage and SMM risk in a high-income country. We conducted a nationwide, population-based cohort study using the Dutch Data InfrAstructure for ParEnts and childRen (DIAPER). The cohort consisted of 832 866 women who gave birth in the Netherlands between 1 January 2012, and 31 December 2021. Multilevel multivariable Poisson regression was used to calculate adjusted risk ratios (aRRs) and 95% confidence intervals (CIs) for individual and neighbourhood-level measures of socioeconomic disadvantage. The role of pre-existing physical and mental health conditions in this association was examined using causal mediation analysis. Individual-level measures of socioeconomic disadvantage showed the strongest association with SMM, with the largest risk ratio of SMM between women with individual low educational attainment compared to those with high educational attainment (aRR 1.41, 95% CI 1.33–1.50), whereas the aRR for low compared to high neighbourhood education was 1.21 (95% CI 1.14–1.28). Physical health conditions mediated between 11% and 29% of the association with SMM across the different measures of disadvantage. Individual measures of socioeconomic position are more strongly associated with SMM than neighbourhood-level measures and pre-existing physical health conditions are important factors in this association. Future research should recognize the potential underestimation of risk when using neighbourhood-level disadvantage as a proxy for individual disadvantage.

## Introduction

In high-income countries, socioeconomic disadvantage is consistently associated with an increased risk of maternal mortality and severe maternal morbidity (SMM). A recent meta-analysis of 52 studies [1] reported pooled odds ratios ranging from 1.29 to 1.48 for socioeconomic disadvantage and SMM and 1.61 to 2.10 for maternal mortality, depending on how socioeconomic disadvantage was measured.

However, due to database limitations, many studies use neighbourhood-level socioeconomic position rather than the individual measure. Although neighbourhood socioeconomic disadvantage may exert unique health effects through environmental exposures such as air pollution and access to green space and services, it may not accurately capture individual socioeconomic circumstances, including individual financial resources, literacy levels, responsiveness to health messages, chronic stress, or occupational exposures [2]. No study has directly compared the impact of individual- versus neighbourhood-level disadvantage on maternal health outcomes [1], leaving both the extent of misclassification bias of using a neighbourhood measure as a proxy for individual disadvantage, or the combined effect of individual and neighbourhood disadvantage in this context unknown.

In addition to socioeconomic disparities, women from minoritized ethnic backgrounds and migrant women living in Europe have increased risk of SMM and maternal mortality [3–5]. In many high-income countries, these groups are also more likely to experience socioeconomic disadvantage [4, 5]. However, there is limited evidence for the intersection of socioeconomic disadvantage, ethnicity, and migration status in increasing SMM risk.

Poor preconception health is associated with both socioeconomic disadvantage and an increased risk of SMM and mortality [6, 7]. Women from lower socioeconomic backgrounds are more likely to enter pregnancy with pre-existing health conditions, higher stress levels, and reduced access to preventive healthcare, all of which may contribute to adverse maternal outcomes [8, 9]. However, the extent to which poor preconception health mediates the relationship between socioeconomic disadvantage and SMM has not been quantified.

Therefore, this study aimed to address these gaps by comparing the associations between individual- and neighbourhood-level measures of socioeconomic disadvantage and SMM risk at childbirth in the Netherlands and examining whether these relationships differed across ethnic and migrant groups. A secondary aim was to explore the role of preconception mental and physical health factors in mediating these associations that could serve as key targets for public health interventions.

## Methods

### Study design

A retrospective nationwide population-based cohort study was conducted using the Dutch Data InfrAstructure for ParEnts and childRen (DIAPER) database. Patient and public involvement in the study from a similar high-income country, England, is described in **Box S1**.

### Database

The data sources used for this study were Perined, the national Dutch Hospital Discharge (DHD) register, and the System of Social Statistical Datasets (SSD) linked at the individual level within the nationwide Data-Infrastructure for Parents and Children (DIAPER) [10] (Fig. S1). Additional information is provided in the Supplementary Material.

### Population, exposure, comparison, and outcome

The study population comprised women who had given birth in hospital or at home between 1 January 2013 and 31 December 2021, aged 10–55, and with a gestational age at childbirth ≥20 weeks in the Netherlands. Women were excluded if they had missing information on neighbourhood-level socioeconomic disadvantage scores. If a woman had two or more separate births during the study period, one of the births was randomly selected to avoid within-individual clustering.

Eight different exposures were used to describe socioeconomic disadvantage measured at the time of birth: five neighbourhood-level and three individual-level. The neighbourhood-level measures included Socioeconomic Status Scores for Districts and Neighbourhoods (SES-WOA) composite, SES-WOA Welfare, SES-WOA Employment, SES-WOA Education [11], all grouped into quintiles, and neighbourhood Liveability (LBM 3.0) [12] divided into five groups based on the Netherlands Housing Survey (WoON) 2018 classes [13]. The individual-level measures included Household Disposable Income (<€20 000, €20–39 999, €40–59 999, €60–79 999, and €80–100 000), Individual Educational Attainment (low, medium, and high), and Individual Employment Status (employed/unemployed). For each neighbourhood-level measure, the reference was the least disadvantaged group with the highest SES-WOA or LBM 3.0 scores. For the individual measures, the reference groups were women with a household disposable income of €80–100 000, women with the highest educational level, and employed women.

SMM was defined using the conditions encompassed in the modified English Maternal Morbidity Outcome Indicator (EMMOI) [14, 15], which was modified again to align with the Dutch data (Table S1). The DIAPER database does not contain OPCS-4 procedure codes; therefore, the definition was limited to diagnoses of ICD-10 codes only. Based on existing literature and clinical knowledge, a directed acyclic graph (DAG) (Fig. S2) was constructed *a priori* to conceptually represent confounders and mediators.

Covariates included age (in 5-year categories except for those younger than 20 or older than 40, who were grouped into single categories), ethnicity (collapsed into Caucasian, Mediterranean [Turkish and North African], Other African, Other Asian and Other including Mixed), migration status: Dutch native (yes/no), primiparous (yes/no), and year of the birth included as a categorical variable. The mediators included were history of pre-existing medical condition (yes/no) and history of pre-existing mental health problems (yes/no), for which information was based on ICD-9 codes (2000–13) and ICD-10 codes (2013 onwards) (Table S2):

### Statistical analysis

Statistical analysis was performed using Stata version 16. Statistical significance was assumed to be a two-tailed *P* values of less than .05. The characteristics of women are presented as numbers and percentages by SES-WOA. The individual measures are presented by the corresponding neighbourhood measure to describe the proportion of women who lived in the quintile corresponding to the neighbourhood measure in each individual measure. Multi-level Poisson regression models with a random intercept were used to calculate the risk ratios (and 95% confidence interval [CI]) of SMM for the neighbourhood-level measures to account for clustering between individuals who shared the same postcode (PC4). The intraclass coefficient (ICC) was used to quantify the proportion of the total variance that is due to differences between individuals compared to differences between groups. A fixed effects Poisson regression model was used to calculate the risk ratios (and 95% CI) of SMM for individual measures. A complete case analysis was used for all models, and the reference category was the least disadvantaged group that had the highest socioeconomic advantage. Table S3 describes the covariates adjusted for in each exposure in each model. For the composite SES-WOA, using Model 1 risk ratios were calculated separately for the major components of the composite SMM outcomes in the most disadvantaged SES-WOA quintile compared to the least.

It was planned *a priori* to test for effect modification between each measure of disadvantage and ethnicity and each neighbourhood-level measure with its corresponding individual-level measure. If there was evidence of effect modification based on likelihood ratio test (LRT), all ethnic groups were compared to a standard reference category (the least disadvantaged Caucasian women category) to provide a consistent baseline across all comparisons. Absolute measures were then calculated using average adjusted predictive margins for each ethnicity category and socioeconomic disadvantage. Univariable and multivariable (using Model 1) population attributable fractions (PAFs) (and their 95% CI) were calculated using the *punaf* command in Stata for each measure of disadvantage. The reference group was women in each highest socioeconomic advantage group, those from a Caucasian ethnic background in the highest socioeconomic group, and finally, those from a Caucasian ethnic group who were Dutch natives in the highest socioeconomic group. These reference groups were selected to enable meaningful comparisons by representing the baseline risk within the population, starting with the most socioeconomically disadvantaged group, and progressively adjusting for ethnicity and nativity, culminating in the group with the presumed lowest risk. The rate in the reference group was then applied to the women in the entire cohort.

The *paramed* command in Stata was used to examine the role of selected mediators (pre-existing physical and mental health conditions) on the association between socioeconomic disadvantage and SMM (Table S4). Logistic and Poisson regression models were used to estimate odds ratios and risk ratios for the mediator and outcome models, respectively, since the *paramed* command does not support Poisson regression for the mediator model. The exposure compared the lowest to the highest measure of disadvantage.

### Sensitivity analyses

A sensitivity analysis was conducted by creating a new ethnicity variable further stratified by migration status, where each ethnic group was divided based on whether individuals were born in the Netherlands (yes/no). Model 1 was tested for evidence of effect modification between the measures of socioeconomic disadvantage and this new variable using the LRT. Each ethnic group was then compared to a standard reference point (least disadvantaged Caucasian-Dutch natives in the highest category of socioeconomic advantage). A second sensitivity analysis involved re-categorizing the neighbourhood-level variables to be the same size (percent of the population) as their corresponding individual-level measure. This was done to ensure comparability between individual- and neighbourhood-level effects and to assess whether differences in category size influenced the observed associations.

### Missing data

For the individual-level exposures, sensitivity analysis was conducted by imputing the values for the missing values of the exposures, using fully conditional specification multiple imputations by chained equations (MICE) to generate 50 datasets. As less than 5% of the data on the other covariates was missing, these values were not imputed.

## Results

### Descriptive statistics

The cohort comprised 832 866 women who gave birth from 1 January 2013 to 31 December 2021. The identification of the study population is shown in Fig. S3. The overall incidence of SMM was 16.1 per 1000 maternities (95% CI 15.8–16.4). Women who lived in the most disadvantaged areas were more likely to be younger, from minoritized ethnicity groups, to be obese and have a history of smoking and/or substance misuse and were less likely to be Dutch natives (Table S5). Sepsis accounted for the largest proportion of SMM (40%) followed by embolism (9%) and uterine rupture (8%) (Fig. S4).

### Correlation between individual and neighbourhood-level measures

Across all socioeconomic disadvantage measures, many women with the highest individual-level socioeconomic advantage lived in the most advantaged neighbourhoods (Table S6). However, the correlation between each individual measure and its corresponding neighbourhood-level measure remained weak (Table S7).

### Multi-level modelling

Women in the study were from 8975 different neighbourhoods with an average of 104.8 maternities per neighbourhood (range: 1–2311). Between-neighbourhood variation accounted for 1.6% (95% CI 1.3%–2.1%) of the variance whereas the remaining 98.4% (95% CI 97.9%–98.7%) was due to between individuals’ variation within neighbourhoods.

### Univariable and multivariable analysis

**Figure 1.**
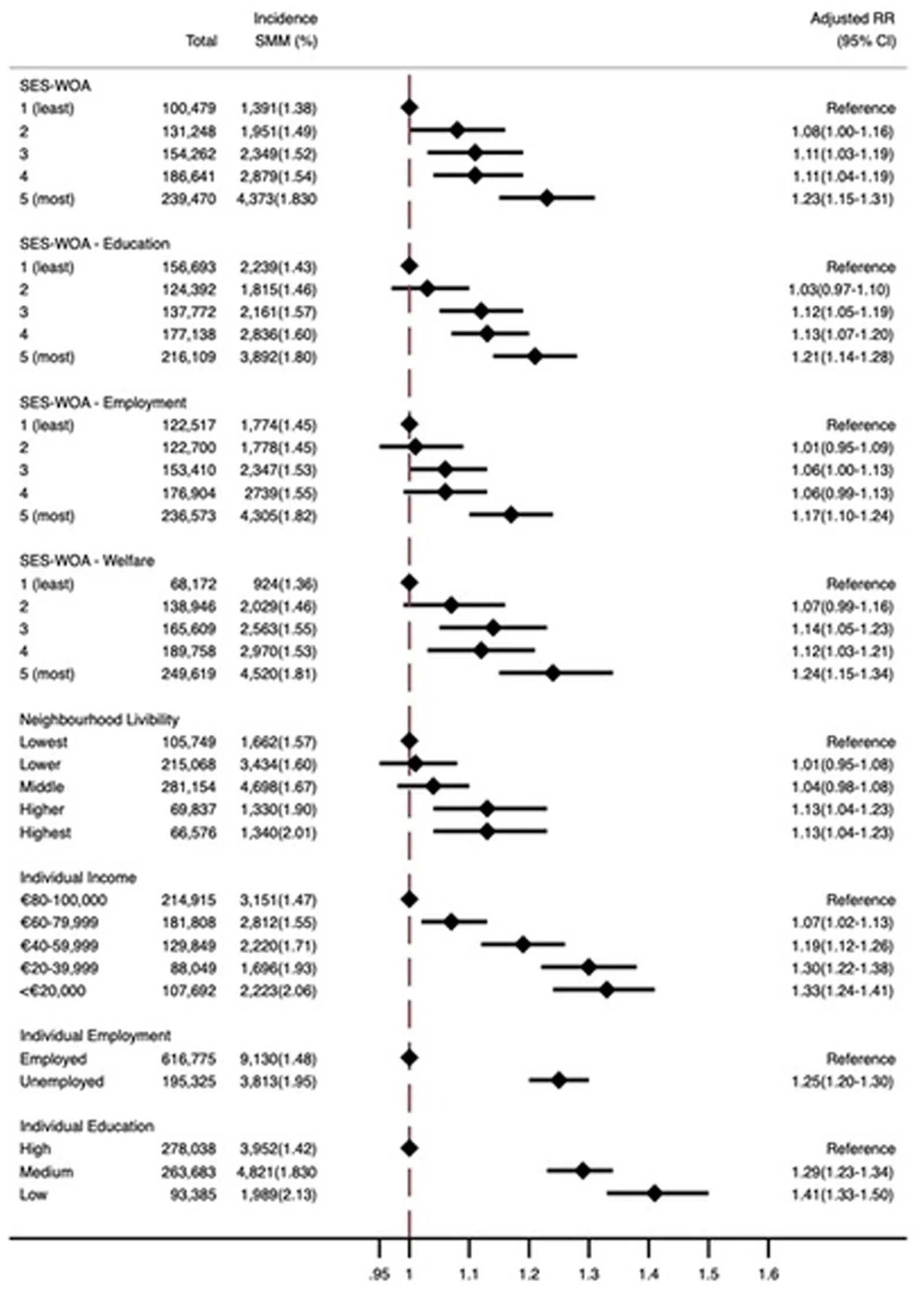
The adjusted risk ratio (RR) and 95% confidence interval (95% CI) of severe maternal morbidity (SMM) in each category of disadvantage measures compared to the least disadvantage category. Least: least disadvantaged quintile; most: most disadvantaged quintile.

Tables S8–S15 show the results of the univariable and multivariable analysis. In the adjusted model, there was a dose–response relationship between increasing neighbourhood SES-WOA Education disadvantage, lower Household Disposable Income, and lower Individual Educational Attainment, and SMM, with the strongest association seen between women with low compared to high Individual Educational Attainment and SMM (adjusted risk ratio [aRR] 1.41 [95% CI 1.33–1.55]) (Fig. 1). There was a dose–response relationship between the increasingly disadvantaged SES-WOA quintile and acute cardiac events, embolic events and pancreatitis, with risk ratios of 1.71 (95% CI 1.32–2.23), 1.21 (95% CI 1.14–1.28), and 1.67 (95% CI 1.30–2.15) in the most compared to the least disadvantaged quintile respectively (Fig. S5). Although the risk ratios attenuated after additionally adjusting for individual-level disadvantage measures (Tables S9–S13, Model 3), an association remained between the neighbourhood-level measures and SMM. There were only minor reductions in the risk ratios for the individual-level measures of disadvantage and SMM after adjusting for neighbourhood-level measures (Tables S14–S16, Model 3).

### Effect modification

There was insufficient evidence for effect modification between the neighbourhood-level measures and their corresponding individual-level measures (LRT test *P* values >.05). However, ethnicity modified the association between Household Disposable Income (*P* = .03), Individual Employment (*P* = .02) and SES-WOA Education (*P* = .045) and SMM. Being unemployed increased the risk of SMM across all ethnic groups, but specifically for unemployed Mediterranean (Turkish and North African) women who had a 3.0% (95% CI 2.5%–3.5%) probability of SMM compared to employed Mediterranean women (2.2% (95% CI 1.9%–2.6%)) (Fig. S6). A dose–response relationship was demonstrated for Caucasian women with increased probability of SMM with decreasing Household Disposable Income but no dose–response relationship was seen for minoritized ethnic women (Fig. S7). For SES-WOA Education and SMM, there was a small dose–response among Caucasian women with an increased risk of SMM with decreased average neighbourhood education, but not among women from minoritized ethnic groups (Fig. S8). The number of women, the incidence (%) of SMM and the aRRs (and 95% CI) in each of these measures compared to the least disadvantaged group stratified by ethnicity are shown in Tables S17–S19.

### Population attributable fractions

Across the different measures of disadvantage, Individual Educational Attainment had the highest adjusted PAFs (15.3%, 95% CI 13.0%–17.5%). About 19% (95% CI 17.1%–21.7%) could be attributed to individual educational attainment and ethnic inequalities, and 21.3% (95% CI 18.8%–23.7%) to individual educational attainment and ethnic- and migration-related inequalities (Table 1).

**Table 1.** Univariable and multivariable population attributable fractions (PAFs) and their 95% confidence intervals (95% CIs) of the different measures of socioeconomic disadvantage using the least disadvantaged group of women across all ethnicities, the least disadvantaged group of Caucasian women, and the least disadvantaged group of Caucasian Dutch native women as the reference group

Category	Unadjusted PAF% (95% CI)	Adjusted PAF % (95% CI)
Reference group: the least disadvantaged women (all ethnicities)		
Lowest educational attainment	16.3 (14.2–18.3)	15.3 (13.0–17.5)
Unemployed	7.2 (6.2–8.2)	5.6 (4.5–6.7)
Lowest disposable income	12.4 (9.7–14.9)	12.0 (9.1–14.8)
Lowest liveability neighbourhood	6.4 (1.8–10.8)	5.7 (0.5–10.5)
Live in the lowest SES-WOA Employment neighbourhoods	9.4 (5.3–13.3)	6.8 (2.4–11.0)
Live in the lowest SES-WOA Education neighbourhoods	11.2 (7.5–14.7)	9.9 (6.1–13.5)
Live in the lowest SES-WOA Welfare neighbourhoods	12.4 (8.8–19.6)	12.2 (6.3–17.6)
Live in lowest SES-WOA neighbourhoods	13.0 (8.4–17.3)	11.1 (6.3–15.6)
Reference group: the least disadvantaged Caucasian women		
Caucasian and have the lowest educational attainment	18.6 (16.4–20.8)	19.4 (17.1–21.7)
Caucasian and unemployed	10.7 (9.4–12.0)	11.8 (10.3–13.2)
Caucasian and the lowest household disposable income quintile	14.0 (11.2–16.8)	16.8 (13.9–19.7)
Caucasian and live in the lowest liveability neighbourhoods	8.4 (3.6–13.1)	9.2 (4.2–13.9)
Caucasian and live in in lowest SES-WOA Employment neighbourhoods	12.3 (7.9–16.6)	12.7 (8.3–17.0)
Caucasian and live in lowest SES-WOA Education neighbourhoods	13.6 (9.7–17.4)	14.7 (10.8–18.5)
Caucasian and live in lowest SES-WOA Welfare neighbourhoods	16.4 (10.6–21.8)	17.2 (11.4–22.6)
Caucasian and live in lowest SES-WOA neighbourhoods	16.1 (11.2–20.7)	16.7 (11.9–21.3)
Reference group: the least disadvantaged Caucasian Dutch-native women		
Caucasian Dutch native and have the lowest educational attainment	13.9 (9.9–17.7)	21.3 (18.8–23.7)
Caucasian Dutch native and unemployed	11.5 (7.0–15.8)	10.9 (9.3–12.4)
Caucasian Dutch native and the lowest household disposable income quintile	15.6 (10.8–20.3)	16.8 (13.6–19.8)
Caucasian Dutch native and live in the lowest liveability neighbourhoods	10.1 (4.9–15.0)	9.6 (4.4–14.5)
Caucasian Dutch native and live in the lowest SES-WOA Employment neighbourhoods	11.5 (6.8–16.0)	11.1 (6.6–15.4)
Caucasian Dutch native and live in the lowest SES-WOA Education neighbourhoods	15.8 (11.5–19.8)	15.7 (11.6–19.6)
Caucasian Dutch native and live in the lowest SES-WOA Welfare neighbourhoods	14.8 (8.6–20.6)	14.2 (8.1–19.9)
Caucasian Dutch native and live in the lowest SES-WOA neighbourhood	16.0 (10.9–21.7)	15.4 (10.2–20.3)

The multivariable analysis adjusted for year, parity, and age (and migration status and ethnicity when comparing to the least disadvantaged women across all ethnicities and migration status’).

### Causal mediation analysis

There was only evidence of mental health problems mediating the effects of neighbourhood Liveability and Individual Educational Attainment and SMM, with 10% (95%CI 1.0%–19.0%) and 7% (95%CI 1.0%–13.0%) of the total effects mediated, respectively (Table 2). The mediation effects of pre-existing physical health conditions were higher and seen across all measures of disadvantage, with the greatest proportion mediated between neighbourhood Liveability and SMM (29% of the total effect (RR: 1.17 [95% CI 1.07–1.27]) and Individual Educational Attainment and SMM where 25% of the total effect (RR: 1.51 [95% CI 1.42–1.60]) were mediated by pre-existing physical health conditions (Table 3).

**Table 2.** Causal mediation analysis of the association between the different measures of socioeconomic disadvantage and severe maternal morbidity (SMM) mediated by mental health problems

Category	Household Disposable Income	Educational Attainment	Employment	SES-WOA	SES-WOA Employment	SES-WOA Welfare	SES-WOA Education	Liveability
**Outcome model**	**Risk ratio** **(95% CI)**	**Risk ratio** **(95% CI)**	**Risk ratio** **(95% CI)**	**Risk ratio** **(95% CI)**	**Risk ratio** **(95% CI)**	**Risk ratio** **(95% CI)**	**Risk ratio** **(95% CI)**	**Risk ratio** **(95% CI)**

Measure of disadvantage^a^	1.34 (1.25–1.44)	1.38 (1.29–1.48)	1.20 (1.15–1.26)	1.27 (1.18–1.36)	1.20 (1.12–1.28)	1.28 (1.18–1.39)	1.20 (1.13–1.27)	1.12 (1.02,1.23)
Mental health problem	1.37 (1.23–1.52)	1.34 (1.22–1.48)	1.33 (1.25–1.41)	1.59 (1.38–1.84)	1.55 (1.36–1.75)	1.59 (1.34–1.89)	1.33 (1.18–1.51)	1.53 (1.33–1.76)
Interaction^b^	1.12 (0.97–1.30)	1.16 (1.01–1.33)	1.23 (1.11–1.35)	0.91 (0.78–1.07)	0.91 (0.78–1.07)	0.92 (0.76–1.11)	1.14 (0.98–1.32)	1.06 (0.87–1.29)

**Mediator model**	**Odds ratio** **(95% CI)**	**Odds ratio** **(95% CI)**	**Odds ratio** **(95% CI)**	**Odds ratio** **(95% CI)**	**Odds ratio** **(95% CI)**	**Odds ratio** **(95% CI)**	**Odds ratio** **(95% CI)**	**Odds ratio** **(95% CI)**

Mental health problem	1.65 (1.61–1.70)	1.89 (1.84–1.94)	1.34 (1.32–1.37)	1.22 (1.18–1.25)	1.07 (1.04–1.10)	1.19 (1.6–1.23)	1.28 (1.25–1.31)	1.33 (1.28–1.38)

**Mediation**	**Risk ratio** **(95% CI)**	**Risk ratio** **(95% CI)**	**Risk ratio** **(95% CI)**	**Risk ratio** **(95% CI)**	**Risk ratio** **(95% CI)**	**Risk ratio** **(95% CI)**	**Risk ratio** **(95% CI)**	**Risk ratio** **(95% CI)**

CDE	1.34 (1.25–1.44)	1.38 (1.29–1.48)	1.20 (1.15–1.26)	1.27 (1.18–1.36)	1.11 (1.03–1.19)	1.28 (1.18–1.39)	1.20 (1.13–1.27)	1.12 (1.02–1.23)
NDE	1.36 (1.27–1.45)	1.40 (1.32–1.49)	1.23 (1.18–1.29)	1.25 (1.17–1.33)	1.10 (1.02–1.17)	1.36 (1.27–1.45)	1.22 (1.15,1.28)	1.13 (1.04–1.23)
NIE	1.02 (1.01–1.02)	1.03 (1.02–1.04)	1.02 (1.01–1.02)	1.01 (1.01–1.01)	1.00 (1.00–1.00)	1.02 (1.01–1.03)	1.01 (1.01–1.01)	1.01 (1.01–1.02)
TE	1.39 (1.30–1.49)	1.45 (1.36–1.54)	1.25 (1.20–1.31)	1.26 (1.18–1.34)	1.10 (1.03–1.17)	1.28 (1.19–1.38)	1.23 (1.16–1.30)	1.15 (1.05–1.25)

**Proportion mediated**	**% Total Effect** **(95% CI)**	**% Total Effect** **(95% CI)**	**% Total Effect** **(95% CI)**	**% Total Effect** **(95% CI)**	**% Total Effect** **(95% CI)**	**% Total Effect** **(95% CI)**	**% Total Effect** **(95% CI)**	**% Total Effect** **(95% CI)**

	6.0 (−1.0–13.0)	7.0 (1.0–13.0)	6.0 (−2.0–10.0)	3.0 (−3.0–9.0)	3.0 (−3.0–9.0)	2.0 (−5.0–9.0)	5.0 (−1.0–11.0).	10.0 (1.0–19.0)

a E.g. Household disposable income, individual educational attainment, etc.

b Interaction between measure of disadvantage and pre-existing mental health conditions.

CDE: controlled direct effects; NDE: natural direct effects; NIE: natural indirect effects; TE: total effects.

**Table 3.** Causal mediation analysis of the association between the different measures of socioeconomic disadvantage and severe maternal morbidity (SMM) mediated by pre-existing physical health conditions (including ischaemic heart disease, hypertension, and diabetes)

Category	Household Disposable Income	Individual Educational Attainment	Employment Status	SES-WOA	SES-WOA Employment	SES-WOA Welfare	SES-WOA Education	Liveability
**Outcome model**	**Risk ratio** **(95% CI)**	**Risk ratio** **(95% CI)**	**Risk ratio** **(95% CI)**	**Risk ratio** **(95% CI)**	**Risk ratio** **(95% CI)**	**Risk ratio** **(95% CI)**	**Risk ratio** **(95% CI)**	**Risk ratio** **(95% CI)**

Measure of disadvantage^a^	1.22 (1.12–1.32)	1.20 (1.10–1.30)	1.14 (1.08–1.2)	1.20 (1.10–1.30)	1.14 (1.06–1.23)	1.23 (1.11–1.35)	1.11 (1.04–1.19)	1.07 (0.96–1.19)
Pre-existing physical health conditions	1.79 (1.66–1.92)	1.81 (1.69–1.93)	1.87 (1.79–1.95)	1.96 (1.76–2.18)	1.95 (1.78–2.15)	1.97 (1.73–2.25)	1.74 (1.59–1.89)	2.03 (1.84–2.25)
Interaction^b^	1.27 (1.13–1.41)	1.32 (1.19–1.48)	1.23 (1.14–1.33)	1.06 (0.94–1.20)	1.07 (0.96–1.19)	1.05 (0.91–1.21)	1.21 (1.09–1.35)	1.09 (0.94–1.27)

**Mediator model**	**Odds ratio** **(95% CI)**	**Odds ratio** **(95% CI)**	**Odds ratio** **(95% CI)**	**Odds ratio** **(95% CI)**	**Odds ratio** **(95% CI)**	**Odds ratio** **(95% CI)**	**Odds ratio** **(95% CI)**	**Odds ratio** **(95% CI)**

Pre-existing physical health conditions	1.53 (1.50–1.56)	1.82 (1.78–1.85)	1.20 (1.19–1.22)	1.22 (1.20–1.24)	1.14 (1.12–1.16)	1.20 (1.18–1.23)	1.32 (1.30–1.34)	1.30 (1.26–1.33)
Mediation								
CDE	1.22 (1.12–1.32)	1.20 (1.1–1.30)	1.14 (1.08–1.2)	1.20 (1.10–1.3)	1.14 (1.06–1.23)	1.23 (1.11–1.35)	1.11 (1.04–1.19)	1.07 (0.96–1.19)
NDE	1.33 (1.25–1.43)	1.34 (1.25–1.42)	1.24 (1.19–1.29)	1.23 (1.16–1.31)	1.17 (1.11–1.24)	1.25 (1.16–1.34)	1.19 (1.13–1.26)	1.11 (1.02–1.21)
NIE	1.08 (1.07–1.09)	1.13 (1.11–1.15)	1.04 (1.03–1.04)	1.03 (1.03–1.04)	1.02 (1.02–1.03)	1.03 (1.03–1.03)	1.05 (1.04–1.05)	1.05 (1.04–1.06)
TE	1.44 (1.35–1.54)	1.51 (1.42–1.60)	1.29 (1.24–1.34)	1.27 (1.19–1.35)	1.20 (1.13–1.27)	1.29 (1.19–1.38)	1.25 (1.18–1.32)	1.17 (1.07–1.27)

**Proportion mediated (%)**	**% Total Effect** **(95% CI)**	**% Total Effect** **(95% CI)**	**% Total Effect** **(95% CI)**	**% Total Effect** **(95% CI)**	**% Total Effect** **(95% CI)**	**% Total Effect** **(95% CI)**	**% Total Effect** **(95% CI)**	**% Total Effect** **(95% CI)**

	19.0 (12.0–26.0)	25.0 (19.0–32.0)	13.0 (9.0–17.0)	12.0 (6.0–18.0)	11.0 (5.0–17.0)	11.0 (4.0–18.0)	19.0 (14.0–25.0)	29.0 (20.0–38.0)

a E.g. Household disposable income, individual educational attainment, etc.

b Interaction between measure of disadvantage and pre-existing physical health conditions.

CDE: controlled direct effects; NDE: natural direct effects; NIE: natural indirect effects; RR: risk ratio; TE: total effects; 95% CI: 95% confidence interval.

### Sensitivity analyses

There was evidence for effect modification by ethnicity and migration status for Individual Educational Attainment (*P* < .001), Individual Employment (*P* < .001) and Household Disposable Income (*P* < .001) and SMM. There was an association between increasing disadvantage across all the disadvantage measures for Caucasian and minoritized ethnic Dutch native-born women, but not for Caucasian and minoritized ethnic non-Dutch native-born women Figs S9–S11. The corresponding risk ratios (95% CI) the incidence of SMM (%) and the total number of women in each ethnic and migrant group are shown in Tables S20–S22.

When the neighbourhood-level measures were recategorized to match their corresponding individual-level measure proportionally, the individual measures continued to have the strongest association with SMM compared to the neighbourhood-level measures (Tables S23–S25).

### Missing data

Table S26 shows the characteristics of the women who had missing data on individual Household Disposable Income, Individual Educational Attainment and neighbourhood Liveability. The results of the multiple imputation of Individual Educational Attainment and Household Disposable Income were not materially different from the complete case analysis (Tables S27 and S28).

## Discussion

This nationwide study of 832 866 maternities from 2013 to 2021 from three linked databases (Perined, DHD, and SSD) showed an overall incidence 16 per 1000 maternities for SMM in the Netherlands. Individual-level measures of socioeconomic disadvantage showed the strongest association with SMM. Among these measures, low educational attainment was associated with the highest risk of SMM, with women in this group experiencing a 41% higher risk compared with those with high educational attainment. Additionally, the intersection of disadvantage, ethnicity, and migration had a varying impact on SMM, contingent on the measure of disadvantage used. Notably, women born in the Netherlands had a clearer dose–response relationship, increased risk for SMM with increasing individual-level disadvantage, than migrant women irrespective of their ethnicity.

These findings have significant implications for understanding the relationship between neighbourhood-level socioeconomic disadvantage and maternal health outcomes in other contexts, such as nationwide confidential inquiries into maternal deaths, which often rely solely on neighbourhood-level measures of disadvantage [1]. Many women are misclassified if neighbourhood-level measures are taken as proxies for individual-level socioeconomic positions, as demonstrated by the findings from the correlation of individual and neighbourhood-level measures. This tends to bias the results towards the null with stronger measure of association for individual measures of disadvantage than the neighbourhood-level measures. The findings from this study contrasts to that of a systematic review [1], which summarized evidence across high-income countries and found no notable differences in the strength of association between individual and neighbourhood-level socioeconomic disadvantage measures and risk of SMM and maternal mortality. However, in this review none of the studies compared individual and neighbourhood-level measures in the same study. Therefore, differences in study design and populations may account for a more similar strength of the association between the two of measures across different studies.

Focusing on women with an individually low socioeconomic position rather than on all women living in the most disadvantaged neighbourhoods may also be a more cost-effective strategy. For example, in the Netherlands, the PAFs were often similar between the neighbourhood-level and individual-level socioeconomic position measures. However, a larger number of women were exposed to neighbourhood-level disadvantage, with over one-fifth of births occurring in the most disadvantaged neighbourhoods. Therefore, many more women in the most disadvantaged areas would need an intervention or a change in their social circumstances, compared to if interventions were targeted at individually disadvantaged women to reduce the same proportion of SMM. Despite the limitations of using neighbourhood-level socioeconomic disadvantage the choice between individual- and neighbourhood-level measures should be guided by the research question. Individual-level measures may be most appropriate when examining personal risk factors or clinical pathways, whereas neighbourhood-level measures are particularly relevant for understanding contextual determinants of SMM and for informing policies or interventions that operate at the neighbourhood level, such as community-based or place-focused approaches.

The role of pre-existing physical and mental health conditions in mediating the relationship between socioeconomic disadvantage and SMM offers valuable insights for policymakers on targeted intervention strategies. In the Netherlands, pre-existing physical health conditions accounted for 11%–29% of the association between socioeconomic disadvantage and SMM, with particularly pronounced effects observed in individual-level measures and neighbourhood liveability. Public health interventions to improve pre-conception health may reduce the incidence and severity of pre-existing physical health conditions for women with lower socioeconomic positions, reducing the disparities highlighted in this study. Preconception care provides a valuable chance to identify, screen, manage and prevent health risk factors, including long-term physical and mental health conditions, alcohol use, smoking, lack of physical activity, poor diet, insufficient social support, and low immunization rates [16]. A study among women with low health literacy in the Netherlands found that most were unaware of preconception counselling but expressed strong interest in participating [17]. Together with findings from our study, this supports the need to prioritize preconception care interventions for higher-risk women, through a multidisciplinary longitudinal approach through various disciplines of healthcare, and to enhance the accessibility and availability of preconception counselling [18].

This study enhances our understanding of the complex interplay between ethnicity, migration status, socioeconomic disadvantage, and SMM risk. Unemployment had a strong association with SMM among Turkish and North African women, whereas household disposable income and neighbourhood education had a weaker association with SMM in minoritized ethnic women compared to Caucasian women. Migration status further modified the association between individual disadvantage and SMM, where the association between disadvantage and SMM was weaker for migrant groups for both Caucasian and minoritized ethnic women, which has also been found in perinatal studies [19–22]. Social and health-related challenges unique to new migrants [23] may heighten SMM risk across the socioeconomic spectrum whereas second-generation migrants may have ‘assimilated’ into the stratification system of the receiving society [24]. These findings highlight the need for a nuanced understanding of the intersection between socioeconomic disadvantage, ethnicity and migration. A one-size-fits-all approach to measuring and addressing these risk factors is inadequate; interventions should consider the overlapping and distinct challenges faced by different groups.

### Strengths and limitations

The strengths and limitations of using a large-population-based administrative database and a composite outcome of SMM have been described elsewhere [4]. A strength of this study is that Perined is representative of the pregnant population in the Netherlands. This study used two further linked databases, including Statistics Netherlands, which contains detailed information on a wide range of socioeconomic characteristics. It provides better understanding of the potential misclassification bias associated with using neighbourhood-level as a proxy for individual-level socioeconomic position measures. The DIAPER database contained detailed information on ethnicity and migration status, which enabled an exploration of the intersection of these factors and the different measures of disadvantage that may aid in informing public health strategies for diverse populations. Finally, the longitudinal nature of this database facilitated the identification of key mediators, such as pre-existing physical and mental health conditions, which enabled examination of mediation effects across different measures of disadvantage.

However, a limitation of is that the database did not include OPCS-4 procedure codes potentially resulting in a higher likelihood of SMM false negatives. Obesity and smoking and substance misuse were also more likely to have been misclassified, as there was a lower prevalence of these conditions than in other studies. Therefore, we did not assess the extent to which these preconception health behaviours mediated the observed associations. The observed lack of correlation between individual- and neighbourhood-level disadvantage measures in the Netherlands may not be generalizable to other countries. Similarly, regional variation within countries should be considered. For example, areas like Amsterdam or the Hauge, which exhibit high socioeconomic diversity within small geographic units, are likely to show weaker correlations between individual and neighbourhood measures compared to less socioeconomically diverse regions of the Netherlands. Finally, there was 11%–22% missing data on individual measures of socioeconomic disadvantage. However, multiple imputation analysis demonstrated robustness of the results despite missing data.

## Conclusion

This study demonstrated that individual measures of socioeconomic position have moderately stronger association with SMM than neighbourhood-level measures. Future research on the role of socioeconomic disadvantage in maternal health outcomes should acknowledge the limitations of using neighbourhood-level measures as proxies for individual-level factors, examine distinct causal pathways for each measure, and consider disaggregated analyses by subgroups to reveal the nuances of socioeconomic inequalities that may exist in adverse pregnancy outcomes such as SMM. Enhancing access to preconception care for high-risk women could be a key intervention in reducing disparities in SMM.

## Supplementary Material

ckag050_Supplementary_Data

## Data Availability

We are unable to share the individual data used for this study as data linkage and analysis was conducted within the highly safeguarded Remote Access (RA) platform of Statistics Netherlands. All data within this platform are pseudonymized to ensure data safety and confidentiality. Access to the data from Perined, Dutch Hospital Data, and Statistics Netherlands can be requested from the relevant parties.
