# Supplementary material for: The association between individual and neighbourhood-level measures of socioeconomic disadvantage and severe maternal morbidity, in the Netherlands, a retrospective cohort study
Source: Eur J Public Health. 2026 Apr 7;36(2):ckag050. doi: 10.1093/eurpub/ckag050 (PMC13064913; doi:10.1093/eurpub/ckag050)
Supplement: ckag050_Supplementary_Data [file ckag050_supplementary_data.docx]

**Data InfrAstructure for ParEnts and childRen (DIAPER) Database**

Perined is the national pregnancy and birth registry, comprising routine care data on >96% of pregnancies in The Netherlands from 2000 onwards, provided by midwifery, general practice, obstetric and pediatric practices[1]. The DHD register contains the principal diagnosis at discharge in the International Classification of Diseases, ninth and 10^th^ revisions (ICD-9 and ICD-10). From 2000 until 2012, the hospital discharge data was registered in the National Medical Registry (LMR), covering approximately 97% of admissions until 2006. After 2006, the coverage decreased to 81% in 2012 [2]. From 2013 onwards, the hospital discharge data was collected in another registry (LBZ), with no missing hospital admissions data [3]. The SSD covers aggregated demographic yearly data at the individual level of the complete Dutch population (e.g. education level-migration background, household income) [4]. Perined, DHD and SSD data were pseudonymised and accessible only in a secure remote access environment in Statistics Netherlands.

**Socioeconomic Status Scores for Districts and Neighbourhoods (SES-WOA)**

The SES-WOA is an area-level socioeconomic score that provides information on an area's average household welfare, education level and labour participation. The smallest sized area, a ‘buurt’ which translates in English to neighbourhood, was chosen for this study as it is more likely to accurately describe the socioeconomic circumstances of an individual compared to measures taken over a larger area. In this study, the composite SES-WOA variable was divided into quintiles based on the final score, with the first quintile being the least disadvantaged and the fifth the most.

**Sub Scores of the SES-WOA**

**SES-WOA Welfare**

To calculate the score, households are divided into deciles based on their financial disadvantage (income and wealth) compared to all households in the Netherlands. Households in the first decile group belong to the 10% of households with the lowest financial wealth. For a small population of the households (<1%), no data on income are known; these households were excluded from the research population. The average score of all the houses in each neighbourhood is used to calculate the overall neighbourhood score. For this study, the neighbourhood score was then grouped into quintiles.

**SES-WOA Education**

The education domain is based on a Labour force survey that covers 12 million people living in the Netherlands. Coverage decreases with age. The educational level of a household is determined by the highest educational level completed by the primary breadwinner and any partner. Because the educational level file does not contain the educational level of all Dutch people, missing values ​​are imputed using multinomial logistic regression[5]. The educational level of a household is given an average score across the neighbourhood. For this study, the scores were then grouped into quintiles.

**SES-WOA Employment**

Recent labour participation is calculated per household using the Regional Employment Survey, which covers the whole population and measures the maximum extent to which the primary breadwinner and any partner have worked in the last four years. The household's employment status was averaged across a neighbourhood-and the scores were then grouped into quintiles for this study.

**Liveability Meter 3.0 (LBM 3.0)**

The LBM 3.0 [6] was developed by the Dutch Ministry of the Interior and Kingdom Relations and is an instrument that, using five dimensions, estimates quality of life at a scale of 100 × 100-meter grids, which participants’ home addresses are matched to. Liveability is “the extent to which the environment fits the requirements and wishes placed on it by humans”. The measure comprised a judgement model, which is how pleasant residents find living in their neighbourhood, and a behavioural model of the housing market, which was merged to arrive at the final score. Both models were based on the correlation between environmental characteristics and criteria specific to each model used to test how people appreciate different aspects of the living environment. The five dimensions include housing stock, facilities, social cohesion, physical environment, nuisance and insecurity, and they are based on many characteristics of the living environment (45 characteristics from 94 indices). To give meaning to the scores, they are converted into classes. These scores are divided into nine classes ('Very poor' to 'Excellent'). For determining the LBM 3.0 class, the distribution of judgments on liveability is classed according to Woon Onderzoek Nederland 2018 [7], which is a comprehensive survey that aims to gather data about housing conditions, preferences, and experiences of Dutch residents. Using the WoON 2018 classes helps with clear interpretation that aligns with the experiences of residents and policymakers. For this study, to provide enough in each category, the classes were collapsed into five groups: very poor and relatively poor, poor and weak, sufficient and reasonably sufficient, good and very good, and excellent.

**Individual Educational Attainment**

The highest level of education at individual-level was provided by Statistics Netherlands, which uses the International Standard Classification of Education. The analysis used three categories: lower, medium and high education levels. The lower education level includes all years of primary education plus the first three years of senior general secondary education (HAVO) and pre-university secondary education (VWO); the various pathways of prevocational secondary education (VMBO), including lower secondary vocational training and assistant’s training (MBO-1). The medium education level includes upper secondary education (HAVO/VWO), basic vocational training (MBO-2), vocational training (MBO-3), and middle management and specialist education (MBO-4). Higher education refers to associate degree programmes-higher education (HBO/WO) Bachelor programmes-4-year education at universities of applied sciences (HBO) master’s degree programmes at universities of applied sciences and research universities (HBO-WO), and doctoral degree programmes at research universities (WO).

**Household Disposable Income**

Household income was provided by Statistics Netherlands and is standardised with the distribution running from -6 to 100,000 euros. This study divided household disposable income into five groups based on the national standardised distribution of <20,000 euros, 20-39,999 euros, 40-59,000 euros, 60-79,000 euros and 80-100,000 euros. The standardised distribution allows income groups to be defined based on a national benchmark, making income comparisons across households fairer and more interpretable.

**Individual Employment**

Individual-level employment variable was derived from Statistics Netherlands' labour force survey, which is cross-checked using administrative data and contains information on whether the woman was employed full-time or part-time (yes/no). For this study, this variable was categorised into employed and unemployed.

**Box S1**. Contribution of the lived experience group

We conducted several focus groups with six women who had recent experience with pregnancy and faced multiple disadvantages in England. The discussions focused on using routine health data to assess socioeconomic disadvantage at both an individual and neighbourhood level. The women were comfortable with having this data collected about them and using it to categorise the level of their disadvantage. They expressed support for using routine data to enhance understanding of how these factors impact pregnancy outcomes. The group also shared personal experiences of socioeconomic disadvantage and how these factors negatively impacted their pregnancies. Additionally, they spoke of experiences of discrimination and disrespect from maternity staff due to their social factors. Despite these challenges, they expressed a desire for their individual sociodemographic information, such as income level and educational attainment, to be shared with healthcare providers, believing that this would better support the care given to women during pregnancy.


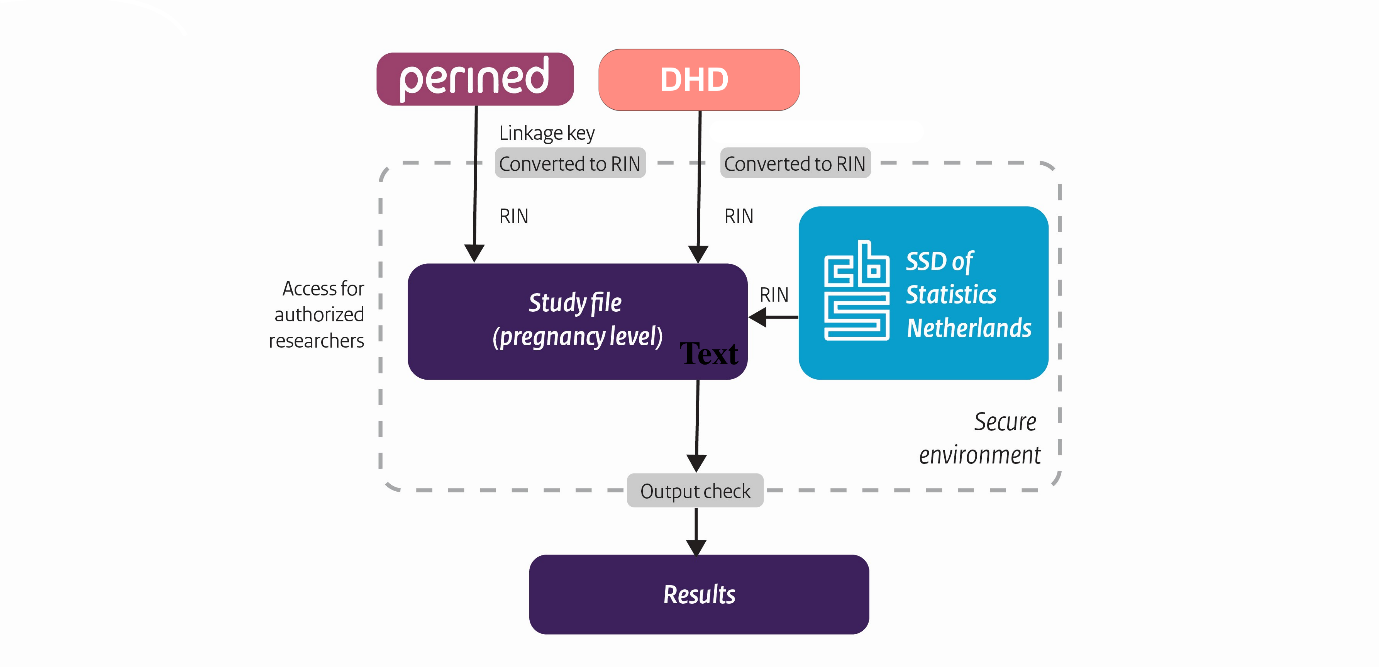


**Figure S1**. The Data-InfrAstructure for ParEnts and children (DIAPER)

**Figure S2.** Direct Acyclic Graph


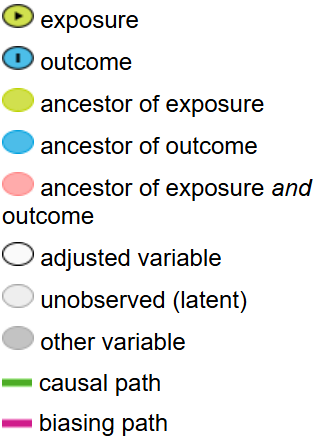

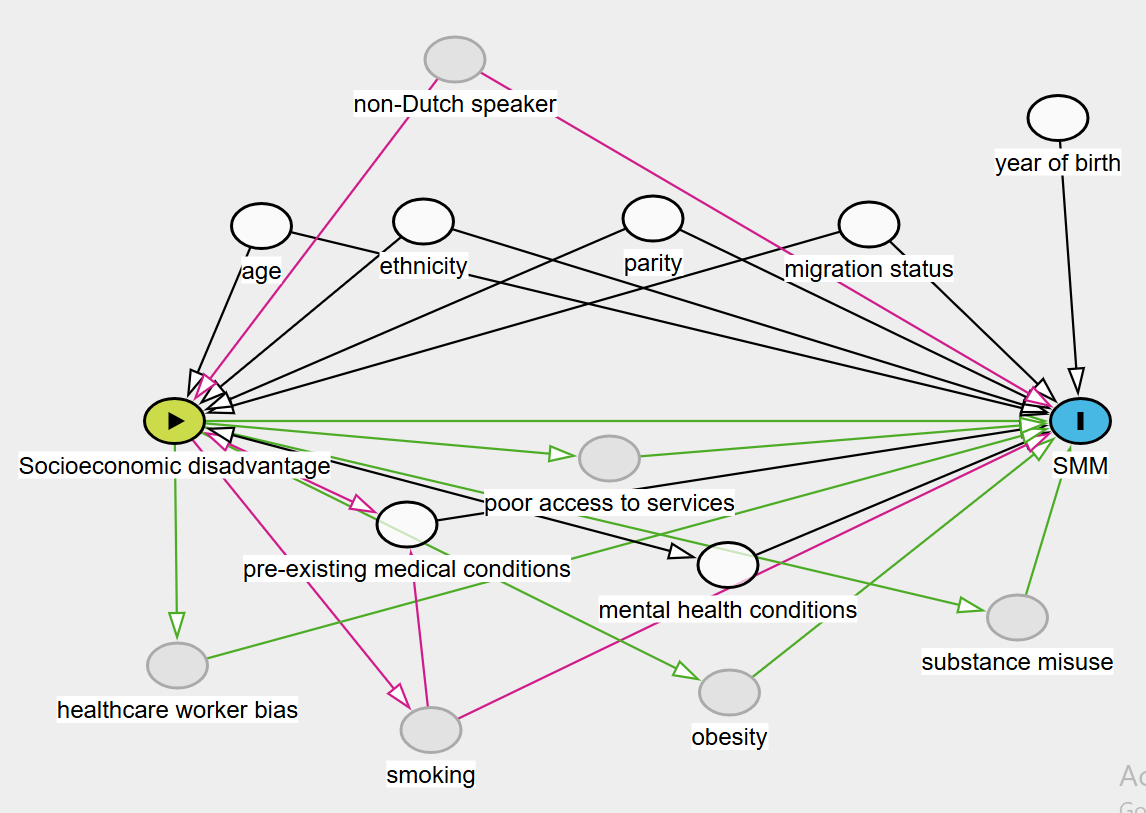


**Table S1.** ICD-10 and OPCS-4 for the modified Dutch Maternal Morbidity Outcome Indicator (DMMOI)[8]. Codes in italics have been removed from the modified English Maternal Morbidity Outcome Indicator as were significantly higher in the DIAPER database than other population-based studies.

| **Morbid event category (ICD-10 codes)** | **Codes** |
| --- | --- |
| Acute abdomen | N733 Female acute pelvic peritonitis  N735 Female pelvic peritonitis, unspecified  K650 Acute peritonitis  K659 Peritonitis, unspecified  *K35 Acute appendicitis*  *K352 Acute appendicitis with generalized peritonitis*  *K353 Acute appendicitis with localized peritonitis*  *K358 Acute appendicitis, other and unspecified*  *K37 Unspecified appendicitis*  K562 Volvulus  K565 Intestinal adhesions (bands) with obstruction  K566 Other and unspecified intestinal obstruction  K593 Megacolon, not elsewhere classified |
| Acute renal failure | O904 Postpartum acute renal failure  N17 Acute renal failure  N170 Acute renal failure with tubular necrosis  N171 Acute renal failure with acute cortical necrosis  N172 Acute renal failure with medullary necrosis  N178 Other acute renal failure  N179 Acute renal failure, unspecified  N19 Unspecified kidney failure  N990 Postprocedural renal failure  I120 Hypertensive renal disease with renal failure  I131 Hypertensive heart and renal disease with renal failure |
| Acute psychosis | F23 Acute and transient psychotic disorders  F230 Acute polymorphic psychotic disorder without symptoms of schizophrenia  F231 Acute polymorphic psychotic disorder with symptoms of schizophrenia  F232 Acute schizophrenia-like psychotic disorder  F238 Other acute and transient psychotic disorders  F239 Acute and transient psychotic disorder, unspecified  F531 Severe mental and behavioural disorders associated with the puerperium, not elsewhere classified |
| Acute cardiac event (cardiac infarction, cardiac failure, cardiomyopathy and cardiac arrest) | O903 Cardiomyopathy in the puerperium  I21 Acute myocardial infarction  I210 Acute transmural myocardial infarction of anterior wall  I211 Acute transmural myocardial infarction of inferior wall  I212 Acute transmural myocardial infarction of other sites  I213 Acute transmural myocardial infarction of unspecified site  I214 Acute subendocardial myocardial infarction  I219 Acute myocardial infarction, unspecified  I42 Cardiomyopathy  I420 Dilated cardiomyopathy  I421 Obstructive hypertrophic cardiomyopathy  I422 Other hypertrophic cardiomyopathy  I423 Endomyocardial (eosinophilic) disease  I424 Endocardial fibroelastosis  I425 Other restrictive cardiomyopathy  I426 Alcoholic cardiomyopathy  I427 Cardiomyopathy due to drugs and other external agents  I428 Other cardiomyopathies  I429 Cardiomyopathy, unspecified  I43* Cardiomyopathy in diseases classified elsewhere  I430* Cardiomyopathy in infectious and parasitic diseases classified elsewhere  I431* Cardiomyopathy in metabolic diseases  I432* Cardiomyopathy in nutritional diseases  I438* Cardiomyopathy in other diseases classified elsewhere  I46 Cardiac arrest  I460 Cardiac arrest with successful resuscitation  I461 Sudden cardiac death, so described  I469 Cardiac arrest, unspecified  I50 Heart failure  I500 Congestive heart failure  I501 Left ventricular failure  I509 Heart failure, unspecified  I110 Hypertensive heart disease with (congestive) heart failure  I119 Hypertensive heart disease without (congestive) heart failure  I130 Hypertensive heart and renal disease with (congestive) heart failure  I132 Hypertensive heart and renal disease with both (congestive) heart failure and renal failure |
| Acute respiratory compromise | J81 Pulmonary oedema  J80 Adult respiratory distress syndrome |
| Cerebral oedema or coma | G936 Cerebral oedema  R402 Coma, unspecified |
| Disseminated intravascular coagulopathy | O450 Premature separation of placenta with coagulation defect (including placental abruption with excessive haemorrhage associated with afibrinoginaemia, disseminated intravascular coagulation, hyperfibrinolysis, hyperfibrinogenaemia)  O460 Antepartum haemorrhage with coagulation defect (antepartum haemorrhage (excessive) associated with afibrinoginaemia, disseminated intravascular coagulation, hyperfibrinolysis, hyperfibrinogenaemia)  O670 Intrapartum haemorrhage with coagulation defect (intrapartum haemorrhage (excessive) associated with afibrinoginaemia, disseminated intravascular coagulation, hyperfibrinolysis, hyperfibrinogenaemia)  D65 Disseminated intravascular coagulation (defibrination syndrome) |
| Cerebrovascular accident | I60 Subarachnoid haemorrhage  I600 Subarachnoid haemorrhage from carotid siphon and bifurcation  I601 Subarachnoid haemorrhage from middle cerebral artery  I602 Subarachnoid haemorrhage from anterior communicating artery  I603 Subarachnoid haemorrhage from posterior communicating artery  I604 Subarachnoid haemorrhage from basilar artery  I605 Subarachnoid haemorrhage from vertebral artery  I606 Subarachnoid haemorrhage from other intracranial arteries  I607 Subarachnoid haemorrhage from intracranial artery, unspecified  I608 Other subarachnoid haemorrhage  I609 Subarachnoid haemorrhage, unspecified  I61 Intracerebral haemorrhage  I610 Intracerebral haemorrhage in hemisphere, subcortical  I611 Intracerebral haemorrhage in hemisphere, cortical  I612 Intracerebral haemorrhage in hemisphere, unspecified  I613 Intracerebral haemorrhage in brain stem  I614 Intracerebral haemorrhage in cerebellum  I615 Intracerebral haemorrhage, intraventricular  I616 Intracerebral haemorrhage, multiple localized  I618 Other intracerebral haemorrhage  I619 Intracerebral haemorrhage, unspecified  I62 Other nontraumatic intracranial haemorrhage  I620 Subdural haemorrhage (acute)(nontraumatic)  I621 Nontraumatic extradural haemorrhage  I629 Intracranial haemorrhage (nontraumatic), unspecified  I63 Cerebral infarction  I630 Cerebral infarction due to thrombosis of precerebral arteries  I631 Cerebral infarction due to embolism of precerebral arteries  I632 Cerebral infarction due to unspecified occlusion or stenosis of precerebral arteries  I633 Cerebral infarction due to thrombosis of cerebral arteries  I634 Cerebral infarction due to embolism of cerebral arteries  I635 Cerebral infarction due to unspecified occlusion or stenosis of cerebral arteries  I638 Other cerebral infarction  I639 Cerebral infarction, unspecified  I64 Stroke, not specified as haemorrhage or infarction |
| Major complications of anaesthesia | O740 Aspiration pneumonitis due to anaesthesia during labour and birth  O741 Other pulmonary complications of anaesthesia during labour and birth  O742 Cardiac complications of anaesthesia during labour and birth  O743 Central nervous system complications of anaesthesia during labour and birth  O749 Complication of anaesthesia during labour and birth, unspecified  O890 Pulmonary complications of anaesthesia during the puerperium  O891 Cardiac complications of anaesthesia during the puerperium  O892 Central nervous system complications of anaesthesia during the puerperium  O290 Pulmonary complications of anaesthesia during pregnancy  O291 Cardiac complications of anaesthesia during pregnancy  O292 Central nervous system complications of anaesthesia during pregnancy  O296 Failed or difficult intubation during pregnancy  O293 Toxic reaction to local anaesthesia during pregnancy |
| Embolic event (pulmonary embolism, amniotic fluid embolism, septic embolism and air embolism) | O88 Obstetric embolism  O880 Obstetric air embolism  O881 Amniotic fluid embolism  O882 Obstetric blood-clot embolism  O883 Obstetric pyaemic and septic embolism  O888 Other obstetric embolism  I26 Pulmonary embolism  I260 Pulmonary embolism with mention of acute cor pulmonale  I269 Pulmonary embolism without mention of acute cor pulmonale |
| Shock | R570 Cardiogenic shock  R571 Hypovolaemic shock  R572 Septic shock  R578 Other shock  R579 Shock, unspecified  O751 Shock during or following labour and birth  T805 Anaphylactic shock due to serum  T886 Anaphylactic shock due to adverse effect of correct drug or medicament properly administered  T782 Anaphylactic shock, unspecified  T780 Anaphylactic shock due to adverse food reaction  A483 Toxic shock syndrome |
| Sickle cell anaemia with crisis | D570 Sickle-cell anaemia with crisis |
| Status asthmaticus | J46 Status asthmaticus |
| Status epilepticus | G41 Status epilepticus  G410 Grand mal status epilepticus  G411 Petit mal status epilepticus  G412 Complex partial status epilepticus  G418 Other status epilepticus  G419 Status epilepticus, unspecified |
| Uterine rupture | O710 Rupture of uterus before onset of labour  O711 Rupture of uterus during labour |
| Eclampsia | O15 Eclampsia  O150 Eclampsia in pregnancy  O151 Eclampsia in labour  O152 Eclampsia in the puerperium  O159 Eclampsia, unspecified as to time period |
| Sepsis | O85 Puerperal sepsis  A40 Streptococcal sepsis  A400 Sepsis due to streptococcus, group A  A401 Sepsis due to streptococcus, group B  A402 Sepsis due to streptococcus, group D  A403 Sepsis due to Streptococcus pneumoniae  A408 Other streptococcal sepsis  A409 Streptococcal sepsis, unspecified  A41 Other sepsis  A410 Sepsis due to Staphylococcus aureus  A411 Sepsis due to other specified staphylococcus  A412 Sepsis due to unspecified staphylococcus  A413 Sepsis due to Haemophilus influenzae  A414 Sepsis due to anaerobes  A415 Sepsis due to other Gram-negative organisms  A418 Other specified sepsis  A419 Sepsis, unspecified (including septicaemia)  A327 Listerial sepsis |
| Cerebral venous thrombosis | O873 Cerebral venous thrombosis in the puerperium  I636 Cerebral infarction due to cerebral venous thrombosis, nonpyogenic  I676 Nonpyogenic thrombosis of intracranial venous system (including non-pyogenic thrombosis of cerebral vein and intracranial venous sinus) |
| Acute pancreatitis | K85 Acute pancreatitis  K850 Idiopathic acute pancreatitis  K851 Biliary acute pancreatitis  K852 Alcohol-induced acute pancreatitis  K853 Drug-induced acute pancreatitis  K858 Other acute pancreatitis  K859 Acute pancreatitis, unspecified  K863 Pseudocyst of pancreas |
| Rupture of aortic aneurysm or dissection of aorta | I710 Dissection of aorta (any part)  I711 Thoracic aortic aneurysm, ruptured  I713 Abdominal aortic aneurysm, ruptured  I715 Thoracoabdominal aortic aneurysm, ruptured  I718 Aortic aneurysm of unspecified site, ruptured  I722 Aneurysm and dissection of renal artery  I723 Aneurysm and dissection of iliac artery  I712 Thoracic aortic aneurysm, without mention of rupture  I714 Abdominal aortic aneurysm, without mention of rupture  I716 Thoracoabdominal aortic aneurysm, without mention of rupture  I719 Aortic aneurysm of unspecified site, without mention of rupture |
| Diabetic ketoacidosis | E100 Diabetes mellitus with coma (including hyperglycaemic coma NOS, diabetic coma with or without ketoacidosis, diabetic hyperosmolar coma, diabetic hypoglycaemic coma)  E101 Diabetes mellitus with ketoacidosis |

**Table S2.** International Classification of Diseases, Tenth Revision (ICD-10) and International Classification of Diseases, Ninth Revision (ICD-9) codes for covariates

| **Pre-existing Medical Condition[8]** | **ICD-10[9]** | **Timing** | **ICD-9** |
| --- | --- | --- | --- |
| Diabetes | G590 G632 H280 H360 M142 N083 O240 O241 O243 H350 H352 E103 E113 E123 E133 E143 E10-E14 | Any code from 2003 to start of pregnancy | 250 |
| Heart disease - ischaemic | I252 I20 I21 I22 I23 I24 I25 I270 I272 | Any code from 2003 to start of pregnancy | 410-414 |
| Heart disease – heart failure and cardiomyopathy | I43 I50 I130 I110 I132 | Any code from 2003 to start of pregnancy | 425, 428 |
| Heart disease- congenital | Q2 €20-39,9990-Q2 €20-39,9996 | Any code from 2003 to start of pregnancy | 745, 746, 747 |
| Heart disease – valve | I34  I35  I05-I08 | Any code from 2003 to start of pregnancy |  |
| Hypertension | I10-I13 I15 | Any code from 2003 to start of pregnancy | 401, 402, 403, 404 |
| Thyroid disease | E035 E038 E039 E050 E051 E052 E055 E058 E059 E062 E063 E065 E069 H062 | Any code from 2003 to start of pregnancy | 394, 395, 396, 397 |
| Obstructive lung disorders – cystic fibrosis | E84 | Any code from 2003 to start of pregnancy | 277 |
| Restrictive lung disorders | J60 J61 J62 J63 J64 J65 J66 J67 J84 J841 J701 J703 J704 G532  M633 D86 | Any code from 2003 to start of pregnancy | 500, 501, 502, 503, 504, 504, 505, 515, 516, 508, 3526, 135 |
| Polyarthropothies | I730 J990 M350 M05 M06 M34 L93 M32 | Any code from 2003 to start of pregnancy | 714, 7102, 4330, 7148, 7101, 7100, 6954 |
| Obstructive lung disorders | J45, J46 | Any code from 2003 to start of pregnancy | 493 |
| Inflammatory bowel disease | K51 K50 | Any code from 2003 to start of pregnancy | 555, 556 |
| Coeliac | K900 | Any code from 2003 to start of pregnancy | 5790 |
| Infective Hepatitis | B150 B160 B190 B18 | Any code from 2003 to start of pregnancy | 0700, 0707, 0702, 07032, 0703, 0704, 5714, 5722 |
| Alcohol related liver disease | K701 K702 K703 K704 K70 | Any code from 2003 to start of pregnancy | 5710, 5711, 5712, 5713 |
| Cirrhosis and liver failure | K717 K740 K741 K742 K744 K745 K746 K72 | Any code from 2003 to start of pregnancy | 4561, 5712, 5715, 4560, 5722, 5723, 5724, 7824, 7895, 1550, 4562, 7824, 7895, V427, 5719, |

**Table S2. (Continued)**

| **Pre-existing Medical Condition[8]** | **ICD-10[9]** | **Timing** | **ICD-9** |
| --- | --- | --- | --- |
| Other liver disease | K743 K754 K711 K762 K763 | Any code from 2003 to start of pregnancy | 570, 5730, 5714, 5733 |
| Cerebrovascular disease | I61 I64 I60 I65 I66 I691 I630 I631 I632 I633 I634 I635 I638 I639 I693 G463 G464 G465 G466 G467 G468 I694 I690 G450 G451 G452 G453 G454 G458 G459 G460 G461 G462 | Any code from 2003 to start of pregnancy | 430 - 438 |
| Epilepsy | G40 G41 | Any code from aged 11+ to start of pregnancy | 345 |
| Chronic kidney disease | N185 T824 Y602 Y612 Y841 Z491 Z492 Z992 N165 T861 Z940 N183 N184 N187 N188 N189 N186 | Any code from 2003 to start of pregnancy | 585 |
| Psoriasis and Eczema | l20 l40 M090 | Any code from aged 11+ to start of pregnancy | 696, 692 |
| HIV | F024 B20 B21 B22 B23 B24 R75 Z21 | Any code from 2003 to start of pregnancy | 042, V08 |
| Cancer | C880 C882 C221 C751 C752 C753 C754 C755 C220 C222 C223 C224 C227 C229 C260C261  C268 C269 C300 C301 C380 C381 C382 C383 C384 C388 C390 C398 C399 C750 C758 C759 C883 C884 C887 C889 C797 C795 C784  C785 C793 C794 C787 C780 C781 C783 C788 C790 C791 C792 C796 C798 C799 C786 C782 C81 C91- C95 C82-C85 C86 C90 C40 C41 C70 – C72 C51-C53 C31-C34 C43-C49 C64-C67 C97 C00-C21 C23-C25 C37 C57 C58 C60 C63 C68 C69 C74 C76 C80 C96 C56 C73 C54 C55 C77 C50 D05 | Any code from 2003 to start of pregnancy | 140-239 |
| Transplant | Z94 | Any code from 2003 to start of pregnancy | V42, 5569, 5059, 3350- 3352, 336, 5280, 4697, 9968, 5569, 5059, 3350, 3351, 3352, 336, 5280, 4697, 556 |
| Fibroids | D25 | Any code from 2003 to start of pregnancy | 218 |
| Endometriosis | N80 | Any code from 5 years prior to pregnancy to start of pregnancy | 6170 |

**Table S2. (Continued)**

| **Pre-existing Medical Condition[8]** | **ICD-10[9]** | **Timing** | **ICD-9** |
| --- | --- | --- | --- |
| Inflammatory bowel syndrome | K58 | Any code from 5 years prior to pregnancy to start of pregnancy | 5641 |
| Post viral and related fatigue | G933 | Any code from 5 years prior to pregnancy to start of pregnancy | 7808 |
| Fibromyalgia | M797 | Any code from 5 years prior to pregnancy to start of pregnancy | 7291 |
| Thrombophilia and thromboembolic disease | D68, I74-I76, I26, I80-I82 | Any code from 2003 to start of pregnancy | 4151, 4511, 4512, 4518, 4519, 452, 453, 2898 |
| **Obesity** | E66, Z684 | Any code from 5 years prior to pregnancy up to and including the delivery episode | 2780 |

| **Substance misuse** | **Coded from 5 years prior to index pregnancy up unto the start of pregnancy** | | |
| --- | --- | --- | --- |
| **ICD-10 condition[10]** | **ICD-10** | **ICD-9 condition[11]** | **ICD-9** |
| Mental and behavioural disorders due to psychoactive substance use | F11-F17, F19 (not F171) | Alcohol use and alcohol-related disorders | 2910, 2911, 2912, 2913, 2914, 2915, 2918, 2919, 3030, 3039, 3050, 3575, 4255, 5353, 5353, 5710, 5711, 5712, 5713, E860 |
| Finding of drugs not normally found in blood | R781-R785 | Amphetamines | 3044, 3057 |
| Poisoning by drugs, medicaments and biological substances | T36-T50 (not T506) | Cannabis | 3043, 3052 |
| Poisoning, undetermined intent | Y10-Y14 | Cannabis dependence | 3042, 3056,9685, E938 |
| Drug rehabilitation | Z503 | Cocaine | 3045, 3053, 9696, E8541, E9396 |
| Drug abuse counselling and surveillance | Z715 | Drug-induced mental disorders | 2920, 2921, 2922, 2928, 2929 |
| Drug use | Z722 | Hallucinogens | 3041, 3054, 9696, E854, E969 |
| Personal history of psychoactive substance abuse | Z864 | Opioids | 3040, 3047, 3055, 9650, E8500, E9350 |
| Mental and behavioural disorders due to use of volatile solvents | F18 | Sedatives, hypnotics, anxiolytics, tranquilizers, barbiturates | 3041, 3054 |
| Accidental poisoning by and exposure to noxious substances | X40–X44, X46-X49 | Other | 3046, 3048, 3049, 3059, 6483, V6542 |
| Poisoning by chemical or noxious substance, undetermined intent | Y16-Y19 |  |  |

**Table S2. (Continued)**

| **Substance misuse** | **Coded from 5 years prior to index pregnancy up unto the start of pregnancy** | | |
| --- | --- | --- | --- |
| **ICD-10 condition[10]** | **ICD-10** | **ICD-9 condition[11]** | **ICD-9** |
| Special epileptic syndromes - (related to alcohol, drugs, etc) | G405 |  |  |
| Blood-alcohol and blood-drug test | Z040 |  |  |
| Mental and behavioural disorders due to use of alcohol | F10 |  |  |
| Degeneration of nervous system due to alcohol | G312 |  |  |
| Alcoholic polyneuropathy | G621 |  |  |
| Alcoholic myopathy | G721 |  |  |
| Alcoholic cardiomyopathy | I426 |  |  |
| Alcoholic gastritis | K292 |  |  |
| Alcoholic liver disease | K70 |  |  |
| Alcohol-induced acute pancreatitis | K852 |  |  |
| Alcohol-induced chronic pancreatitis | K860 |  |  |
| Finding of alcohol in blood | R780 |  |  |
| Poisoning: antidotes and chelating agents, not elsewhere classified | T506 |  |  |
| Toxic effect of alcohol | T51 |  |  |
| Accidental poisoning by exposure to alcohol | X45 |  |  |
| Poisoning by exposure to alcohol, undetermined intent | Y15 |  |  |
| Evidence of alcohol involvement determined by blood alcohol level | Y90 |  |  |
| Evidence of alcohol involvement determined by level of intoxication | Y91 |  |  |
| Alcohol rehabilitation | Z502 |  |  |
| Alcohol abuse counselling and surveillance | Z714 |  |  |
| Alcohol use | Z721 |  |  |
| **Mental Health conditions/ behavioural disorders** | **Coded from 5 years prior to index pregnancy to the start of pregnancy** | | |
| **ICD-10 condition[10]** | **ICD-10** | **ICD-9 condition[12]** | **ICD-9** |
| Organic, including symptomatic, mental disorders | F00-F09 | Organic psychotic conditions | 290–294 |
| Schizophrenia, schizotypal and delusional disorders | F20-F29 | Schizophrenic disorders | 295 |
| Mood (affective) disorders | F30-F39 | No schizophrenic psychotic disorders | 297, 298 |
| Neurotic, stress-related and somatoform disorders | F40-F48 | Episodic mood disorders | 296, 311 |
| Behavioural syndromes associated with physiological disturbances and physical factors | F50-F59 | Anxiety, dissociative, and somatoform disorders | 300 |
| Disorders of adult personality and behaviour | F60-F69 | Physiological malfunction arising from mental factors | 306 |

**Table S2. (Continued)**

| **Mental Health conditions/ behavioural disorders** | **Coded from 5 years prior to index pregnancy to the start of pregnancy** | | |
| --- | --- | --- | --- |
| **ICD-10 condition[10]** | **ICD-10** | **ICD-9 condition[12]** | **ICD-9** |
| Mental retardation | F70-F79 | Personality disorders and certain other nonpsychotic mental disorders | 301 |
| Disorders of psychological development | F80-F89 | Mental health diagnoses of childhood | 312-316, 299 |
| Behavioural and emotional disorders with onset usually occurring in childhood and adolescence | F90-F98 | Specific delays in development | 317-319 |
| Unspecified mental disorder | F99 | Unspecified nonpsychotic mental disorder | 307-310 |
| Sedatives, hypnotics and antianxiety drugs | Y47 | Personal history of mental disorder | V11 |
| Psychotropic drugs, not elsewhere classified | Y49 |  |  |
| Personal history of other mental and behavioural disorders | Z865 |  |  |

**Table S3.** Covariates included in each model as confounding factors in the association between different measures of socioeconomic disadvantage and severe maternal morbidity.

|  | **Model 1** | **Model 2** | **Model 3** | **Model 4** |
| --- | --- | --- | --- | --- |
| SES-WOA | age, parity, ethnicity, migration status | Model 1+ pre-existing medical and mental health conditions, substance misuse | Model 1 + neighbourhood liveability | Model 1 + individual educational attainment, household disposable income, individual employment |
| SES-WOA Education | age, parity, ethnicity, migration status | Model 1+ pre-existing medical and mental health conditions, substance misuse | Model 1 + individual educational attainment | Model 1 + individual educational attainment, household disposable income, individual employment |
| SES-WOA Welfare | age, parity, ethnicity, migration status | Model 1+ pre-existing medical and mental health conditions, substance misuse | Model 1 + household disposable income | Model 1 + individual educational attainment, household disposable income, individual employment |
| SES-WOA Employment | age, parity, ethnicity, migration status | Model 1+ pre-existing medical and mental health conditions, substance misuse | Model 1 + individual employment status | Model 1 + individual educational attainment, household disposable income, individual employment |
| Neighbourhood Liveability | age, parity, ethnicity, migration status | Model 1+ pre-existing medical and mental health conditions, substance misuse | Model 1 + SES-WOA | Model 1 + individual educational attainment, household disposable income, individual employment |
| Household Disposable Income | age, parity, ethnicity, migration status | Model 1+ pre-existing medical and mental health conditions, substance misuse | Model 1 + SES-WOA Welfare | NA |
| Individual Educational Attainment | age, parity, ethnicity, migration status | Model 1+ pre-existing medical and mental health conditions, substance misuse | Model 1 + SES-WOA Education | NA |
| Individual Employment Status | age, parity, ethnicity, migration status | Model 1+ pre-existing medical and mental health conditions, substance misuse | Model 1 + SES-WOA Employment | NA |

**Figure S3.** Flow chart showing the identification of the cohort 2013-2021


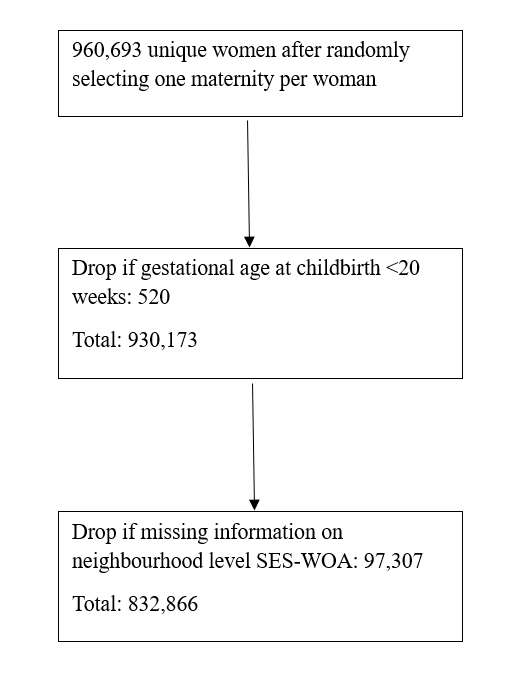


**Table S4.** The breakdown of causal mediation analysis using the *paramed* command

| Controlled Direct Effects (CDE) | The effect of socioeconomic disadvantage on SMM if the mediator is set at a predefined level |
| --- | --- |
| Natural direct effects (NDE) | The effect of the exposure on the outcome not involving the mediator, holding the mediator at a constant level that it would have been without the exposure |
| Natural indirect effects (NIE) | The effect of the exposure on the outcome that operates through the mediator |
| Total effects (TE) | The total effect of socioeconomic disadvantage on SMM (NIE + NDE) |

**Table S5.** Characteristics of the study population stratified by SES-WOA

|  | **Most disadvantaged 20%** | **More disadvantaged 20%-40%** | **Less disadvantaged 40%-60%** | **Less disadvantaged 60%-80%** | **Least disadvantaged 80%-100%** | **Total** |
| --- | --- | --- | --- | --- | --- | --- |
|  | **n (%)** | **n (%)** | **n (%)** | **n (%)** | **n (%)** | **N (%)** |
| **Total** | **245,783**  **(29.5)** | **191,521**  **(23.0)** | **158,269**  **(19.0)** | **134,403**  **(16.1)** | **102,890**  **(12.4)** | **832,866 (100.0)** |
| **SMM** |  |  |  |  |  |  |
| No | 241,231  (98.2) | 188,554  (98.4) | 155,827  (98.5) | 132,401  (98.5) | 101,440  (98.6) | 819,453  (98.4) |
| Yes | 4,552  (1.9) | 2,967  (1.6) | 2,442  (1.5) | 2,002  (1.5) | 1,450  (1.4) | 13,413  (1.6) |
| **Age group** |  |  |  |  |  |  |
| <20 | 3,982  (1.6) | 1,755  (0.9) | 1,049  (0.7) | 724  (0.5) | 333  (0.3) | 7,843  (0.9) |
| 20-25 | 31,066  (12.6) | 17,166  (9.0) | 11,137  (7.0) | 7,150  (5.3) | 3,599  (3.5) | 70,118  (8.4) |
| 25-30 | 77,825  (31.7) | 60,478  (31.6) | 47,919  (30.3) | 37,738  (28.1) | 24,612  (23.9) | 248,572  (29.9) |
| 30-35 | 81,913  (33.3) | 72,504  (37.9) | 63,203  (39.9) | 56,595  (42.1) | 45,253  (44.0) | 319,468  (38.4) |
| 35-40 | 41,040  (16.7) | 32,981  (17.2) | 29,016  (18.3) | 26,938  (20.0) | 24,445  (23.8) | 154,420  (18.5) |
| >40 | 9,934  (4.0) | 6,616  (3.5) | 5,932  (3.8) | 5,243  (3.9) | 4,641  (4.5) | 32,366  (3.9) |
| **Parity** |  |  |  |  |  |  |
| Primiparous | 122,848  (50.0) | 98,377  (51.4) | 77,741  (49.1) | 65,603  (48.8) | 47,489  (46.2) | 412,058  (49.5) |
| Multiparous | 122,935  (50.0) | 93,144  (48.6) | 80,528  (50.9) | 68,800  (51.2) | 55,401  (53.8) | 420,808  (50.5) |
| **Ethnicity** |  |  |  |  |  |  |
| Caucasian | 157,668  (65.8) | 157,983  (84.6) | 137,056  (88.9) | 116,946  (89.1) | 89,631  (89.2) | 659,284  (81.2) |
| Mediterranean (North African + Turkish) | 7,846  (3.3) | 2,071  (1.1) | 1,026  (0.7) | 939  (0.7) | 639  (0.6) | 12,521  (1.5) |
| Other African | 11,897  (5.0) | 3,376  (1.8) | 1,673  (1.1) | 1,139  (0.9) | 692  (0.7) | 18,777  (2.3) |
| Other Asian | 16,658  (7.0) | 7,846  (4.2) | 5,349  (3.5) | 4,771  (3.6) | 4,025  (4.0) | 38,649  (4.8) |
| Other including mixed | 45,401  (19.0) | 15,366  (8.2) | 9,159  (5.9) | 7,454  (5.7) | 5,493  (5.5) | 82,873  (10.2) |
| **Migration background** |  |  |  |  |  |  |
| Dutch native | 124,212  (50.5) | 139,803  (73.0) | 124,926  (78.9) | 106,999  (79.6) | 81,271  (79.0) | 577,211  (69.3) |
| Moroccan | 18,633  (7.6) | 5,076  (2.7) | 2,563  (1.6) | 2,139  (1.6) | 1,575  (1.5) | 29,986  (3.6) |
| Other non-western | 33,436  (13.6) | 14,904  (7.8) | 9,812  (6.2) | 7,458  (5.6) | 5,771  (5.6) | 71,381  (8.6) |
| Other western | 33,308  (13.6) | 22,209  (11.6) | 15,801  (10.0) | 12,903  (9.6) | 10,208  (9.9) | 94,429  (11.3) |
| Surinamese | 9,954  (4.1) | 3,094  (1.6) | 2,040  (1.3) | 1,963  (1.5) | 1,615  (1.6) | 18,666  (2.2) |
| Turkish | 19,029  (7.7) | 4,542  (2.4) | 2,102  (1.3) | 2,114  (1.6) | 1,757  (1.7) | 29,544  (3.6) |
| Netherlands Antilles | 7,211  (2.9) | 1,893  (1.0) | 1,025  (0.7) | 827  (0.6) | 693  (0.7) | 11,649  (1.4) |

**Table S5. (Continued)**

|  | **Most disadvantaged 20%** | **More disadvantaged**  **20%-40%** | **Less disadvantaged 40%-60%** | **Less disadvantaged 60%-80%** | **Least disadvantagd 80%-100%** | **Total** |
| --- | --- | --- | --- | --- | --- | --- |
| **Year** |  |  |  |  |  |  |
| 2013 | 29,582  (12.0) | 23,289  (12.2) | 20,018  (12.7) | 16,491  (12.3) | 12,615  (12.3) | 101,995  (12.2) |
| 2014 | 29,176  (11.9) | 22,162  (11.6) | 18,463  (11.7) | 15,305  (11.4) | 11,581  (11.3) | 96,687  (11.6) |
| 2015 | 26,928  (11.0) | 20,025  (10.5) | 16,393  (10.4) | 13,578  (10.1) | 10,101  (9.8) | 87,025  (10.5) |
| 2016 | 26,766  (10.9) | 19,959  (10.4) | 15,829  (10.0) | 13,348  (9.9) | 10,001  (9.7) | 85,903  (10.3) |
| 2017 | 25,498  (10.4) | 19,385  (10.1) | 15,886  (10.0) | 13,298  (9.9) | 10,297  (10.0) | 84,364  (10.1) |
| 2018 | 25,073  (10.2) | 19,314  (10.1) | 15,799  (10.0) | 13,423  (10.0) | 10,449  (10.2) | 84,058  (10.1) |
| 2019 | 26,962  (11.0) | 20,912  (10.9) | 17,377  (11.0) | 15,098  (11.2) | 11,177  (10.9) | 91,526  (11.0) |
| 2020 | 28,000  (11.4) | 22,910  (12.0) | 18,953  (12.0) | 16,628  (12.4) | 13,101  (12.7) | 99,592  (12.0) |
| 2021 | 27,798  (11.3) | 23,565  (12.3) | 19,551  (12.4) | 17,234  (12.8) | 13,568  (13.2) | 101,716  (12.2) |
| **Substance misuse or smoking** |  |  |  |  |  |  |
| No | 238,830  (97.2) | 186,468  (97.4) | 154,438  (97.6) | 131,405  (97.8) | 100,747  (97.9) | 811,888  (97.5) |
| Yes | 6,953  (2.8) | 5,053  (2.6) | 3,831  (2.4) | 2,998  (2.2) | 2,143  (2.1) | 20,978  (2.5) |
| **Obesity** |  |  |  |  |  |  |
| No | 237,074  (96.5) | 185,061  (96.6) | 153,269  (96.8) | 130,397  (97.0) | 100,028  (97.2) | 805,829  (96.8) |
| Yes | 8,709  (3.5) | 6,460  (3.4) | 5,000  (3.2) | 4,006  (3.0) | 2,862  (2.8) | 27,037  (3.3) |
| **Pre-existing mental health problem** |  |  |  |  |  |  |
| No | 217,903  (88.7) | 169,090  (88.3) | 140,502  (88.8) | 119,961  (89.3) | 91,896  (89.3) | 739,352  (88.8) |
| Yes | 27,880  (11.3) | 22,431  (11.7) | 17,767  (11.2) | 14,442  (10.8) | 10,994  (10.7) | 93,514  (11.2) |
| **Pre-existing medical conditions** |  |  |  |  |  |  |
| No | 176,002  (71.6) | 135,426  (70.7) | 112,949  (71.4) | 96,239  (71.6) | 73,569  (71.5) | 594,185  (71.3) |
| Yes | 69,781  (28.4) | 56,095  (29.3) | 45,320  (28.6) | 38,164  (28.4) | 29,321  (28.5) | 238,681  (28.7) |

**Table S5. (Continued)**

|  | **Most disadvantaged 20%** | **More disadvantaged 20%-40%** | **Less disadvantaged 40%-60%** | **Less disadvantaged 60%-80%** | **Least disadvantaged 80%-100%** | **Total** |
| --- | --- | --- | --- | --- | --- | --- |
| **Pre-existing medical conditions (excluding ischemic heart disease, diabetes and hypertension)** |  |  |  |  |  |  |
| No | 178,755  (72.3) | 137,433  (71.76) | 114,641  (72.4) | 97,682  (72.7) | 74,632  (72.5) | 603,143  (72.4) |
| Yes | 67,028  (27.3) | 54,088  (28.2) | 43,628  (27.6) | 36,721  (27.3) | 28,258  (27.5) | 229,723  (27.6) |
| **Migration status and ethnicity** |  |  |  |  |  |  |
| Caucasian and Dutch native | 118,849  (48.4) | 134,521  (70.2) | 120,403  (76.1) | 103,280  (76.8) | 78,621  (76.4) | 555,674  (66.7) |
| Caucasian and non-Dutch native | 38,819  (15.8) | 23,462  (12.2) | 16,653  (10.5) | 13,666  (10.2) | 11,010  (10.7) | 103,610  (12.4) |
| Minoritised ethnicity and Dutch native | 2,369  (1.0) | 1,991  (1.0) | 1,637  (1.0) | 1,507  (1.1) | 975  (1.0) | 8,479  (1.0) |
| Other including mixed and non-Dutch native | 48,864  (19.9) | 15,741  (8.2) | 9,230  (5.8) | 7,539  (5.6) | 5,797  (5.6) | 87,171  (10.5) |
| Mediterranean and non-Dutch native | 7,806  (3.2) | 2,051  (1.1) | 1,011  (0.6) | 927  (0.7) | 632  (0.6) | 12,427  (1.5) |
| Other African and non-Dutch native | 11,655  (4.7) | 3,284  (1.7) | 1,625  (1.0) | 1,103  (0.8) | 667  (0.7) | 18,334  (2.2) |
| Other Asian and non-Dutch native | 11,108  (4.5) | 5,592  (2.9) | 3,704  (2.3) | 3,227  (2.4) | 2,778  (2.7) | 26,409  (3.2) |
| Missing ethnicity and Dutch native | 2,994  (1.2) | 3,291  (1.7) | 2,886  (1.8) | 2,212  (1.7) | 1,675  (1.6) | 13,058  (1.6) |
| Missing ethnicity and non-Dutch native | 3,319  (1.4) | 1,588  (0.8) | 1,120  (0.7) | 942  (0.7) | 735  (0.7) | 7,704  (0.9) |
| **Birth onset method** |  |  |  |  |  |  |
| Induction | 61,873  (26.2) | 46,807  (25.2) | 37,621  (24.5) | 30,789  (23.6) | 22,601  (22.6) | 199,691  (24.8) |
| Elective Caesarean Birth | 20,121  (8.5) | 15,095  (8.1) | 12,847  (8.4) | 10,614  (8.1) | 8,400  (8.4) | 67,077  (8.3) |
| Spontaneous Birth | 154,092  (65.3) | 123,600  (66.6) | 103,117  (67.1) | 89,048  (68.3) | 68,995  (69.0) | 538,852  (66.9) |

**Table S5. (Continued)**

|  | **Most disadvantaged 20%** | **More disadvantaged 20%-40%** | **Less disadvantaged 40%-60%** | **Less disadvantaged 60%-80%** | **Least disadvantaged 80%-100%** | **Total** |
| --- | --- | --- | --- | --- | --- | --- |
| **Place of childbirth** |  |  |  |  |  |  |
| At home | 20,408  (8.4) | 24,259  (12.7) | 22,868  (14.5) | 19,695  (14.7) | 15,406  (15.0) | 102,636  (12.4) |
| Birth centre | 8,351  (3.4) | 4,533  (2.3) | 3,253  (2.1) | 3,309  (2.5) | 2,309  (2.3) | 21,755  (2.6) |
| Hospital (1st line)* | 24,008  (9.8) | 18,686  (9.81) | 15,671  (9.96) | 13,846  (10.36) | 11,885  (11.61) | 84,096  (10.2) |
| Hospital (2nd line)** | 191,240  (78.4) | 142,901  (75.1) | 115,586  (73.4) | 96,842  (72.4) | 72,795  (71.1) | 619,364  (74.8) |
| En route | 26  (0.0) | 15  (0.0) | 17  (0.0) | 8  (0.0) | 7  (0.0) | 73  (0.0) |
| **Mode of childbirth** |  |  |  |  |  |  |
| Unassisted vaginal birth | 168,322  (71.9) | 133,006  (72.6) | 110,767  (73.1) | 94,303  (73.4) | 73,154  (74.1) | 579,552  (72.8) |
| Assisted vaginal birth | 20,426  (8.7) | 16,617  (9.1) | 13,438  (8.9) | 11,509  (9.0) | 8,469  (8.6) | 70,459  (8.9) |
| Elective caesarean birth | 21,378  (9.1) | 16,047  (8.8) | 13,601  (9.0) | 11,262  (8.8) | 8,883  (9.0) | 71,171  (8.9) |
| Emergency caesarean birth | 23,983  (10.2) | 17,567  (9.6) | 13,673  (9.0) | 11,277  (8.8) | 8,120  (8.2) | 74,620  (9.4) |
| Termination of pregnancy | 56  (0.0) | 56  (0.0) | 47  (0.0) | 53  (0.0) | 44  (0.0) | 256  (0.0) |

*1^st^ line = midwifery and GP led care, 2^nd^ line= obstetrician led care

**Table S6.** The cross-tabulation of each individual measure of socioeconomic disadvantage with the corresponding neighbourhood measure N (%)

| **Individual Measure** | **Neighbourhood measure** | | | | | |
| --- | --- | --- | --- | --- | --- | --- |
|  | **Lowest SES-WOA Education 20%** | **Lower SES-WOA Education 20-40%** | **Lower SES-WOA Education 40-60%** | **High SES-WOA Education 60-80%** | **Highest SES-WOA Education 80-100%** | **Total** |
| **Individual Educational Attainment** | | |  |  |  |  |
| Low | 44,127  (19.9) | 22,201  (12.2) | 12,990  (9.2) | 9,159  (7.2) | 7,368  (4.6) | 96,845  (11.6) |
| Medium | 84,559  (38.2) | 67,311  (37.1) | 47,989  (34.0) | 38,247  (30.0) | 32,146  (20.0) | 270,252  (32.4) |
| High | 41,786  (18.9) | 52,715  (29.0) | 50,373  (35.6) | 53,210  (41.8) | 86,990  (54.1) | 284,074  (34.1) |
| Missing | 51,160  (23.1) | 39,421  (21.7) | 30,135  (21.3) | 26,769  (21.0) | 34,210  (21.3) | 181,695  (21.8) |
| Total | 221,632  (26.6) | 181,648  (21.8) | 141,487 (17.0) | 127,385 (15.3) | 160,714 (19.3) | 832,866 (100.0) |
|  | **Lowest SES-WOA Employment 20%** | **Lower SES-WOA Employment 20-40%** | **Lower SES-WOA Employment 40-60%** | **Higher SES-WOA Employment 60-80%** | **Highest SES-WOA Employment 80-100%** |  |
| **Individual Employment** | |  |  |  |  |  |
| Unemployed | 86,189  (35.5) | 43,262  (23.8) | 30,235  (19.2) | 21,985  (17.5) | 19,028  (15.2) | 200,699  (24.0) |
| Employed | 156,759  (64.5) | 138,409  (76.2) | 127,082 (80.8) | 103,530 (82.5) | 106,387 (84.8) | 632,167  (76.0) |
| Total | 242,948  (29.1) | 181,671  (21.8) | 157,317 (18.9) | 125,515 (15.1) | 125,415 (15.1) | 832,866 (100.0) |
|  | **Lowest SES-WOA Welfare 20%** | **Lower SES-WOA Welfare 20-40%** | **Lower SES-WOA Welfare 40-60%** | **Higher SES-WOA Welfare 60-80%** | **Highest SES-WOA Welfare 80-100%** |  |
| **Household Disposable Income (euros)** | | |  |  |  |  |
| <€20,000 | 62,087  (24.2) | 23,928  (12.3) | 13,821  (8.1) | 8,111  (5.7) | 3,143  (4.5) | 111,090  (11.9) |
| €20-39,999 | 39,562  (15.4) | 22,661  (11.6) | 15,198  (9.0) | 9,483  (6.7) | 3,422  (4.9) | 90,326  (9.7) |
| €40-59,999 | 40,923  (16.0) | 35,222  (18.1) | 28,746  (16.9) | 20,381  (14.3) | 7,736  (11.1) | 133,008  (14.2) |
| €60-79,999 | 42,414  (16.6) | 46,132  (23.7) | 44,500  (26.2) | 37,097  (26.1) | 16,040  (23.0) | 186,183  (19.9) |
| €80-100,00 | 41,258  (16.1) | 45,480  (23.3) | 49,407  (29.1) | 52,121  (36.6) | 32,075  (45.9) | 220,341  (23.6) |
| Missing | 29,863  (11.7) | 21,475  (11.0) | 18,088  (10.7) | 15,096  (10.6) | 7,396  (10.6) | 92,918  (9.9) |
| Total | 256,107  (30.1) | 194,898  (23.3) | 169,760 (20.5) | 142,289 (17.1) | 69,812  (8.4) | 832,866 (100.0) |

**Table S6. (Continued)**

| **Neighbourhood measure** | **Neighbourhood measure** | | | | | |
| --- | --- | --- | --- | --- | --- | --- |
|  | **Lowest SES-WOA Composite 20%** | **Lower SES-WOA Composite 20-40%** | **Lower SES-WOA Composite 40-60%** | **Higher SES-WOA Composite 60-80%** | **Highest SES-WOA Composite 80-100%** | **Total** |
| **Liveability** | |  |  |  |  |  |
| Lowest | 56,463  (22.3) | 6,019  (3.1) | 2,102  (1.3) | 1,298  (1.0) | 694  (0.7) | 66,576  (8.0) |
| Lower | 43,330  (17.6) | 15,384  (8.0) | 5,145  (3.3) | 4,089  (3.0) | 1,889  (1.8) | 69,837  (8.5) |
| Middle | 85,007  (34.6) | 79,411  (41.5) | 51,420  (32.5) | 37,715  (28.1) | 27,601  (26.8) | 281,54  (33.7) |
| Higher | 26,570  (10.8) | 51,412  (26.8) | 54,326  (34.6) | 46,832  (34.8) | 35,424  (34.4) | 215,068  (25.8) |
| Highest | 7,665  (3.1) | 16,993  (8.8) | 54,830  (16.5) | 29,136  (21.7) | 25,910  (25.2) | 105,749  (12.7) |
| Missing | 26,748  (10.9) | 22,362  (11.7) | 26,105  (11.8) | 15,333  (11.4) | 11,372  (11.1) | 94,483  (11.5) |
| Total | 245,783  (29.5) | 191,521  (23.0) | 158,269 (19.0) | 134,403 (16.1) | 102,890  (12.4) | 832,866 (100.0) |

**
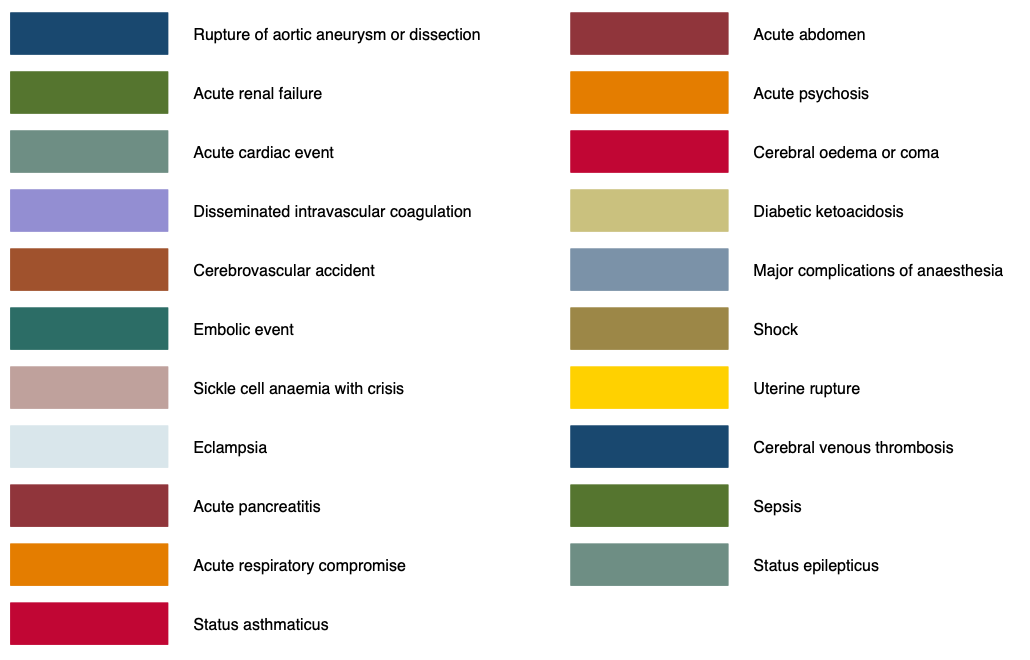
**

**Figure S4.** The proportion (%) of each severe maternal morbidity (SMM) diagnoses (ICD-10) of the final composite outcome

**
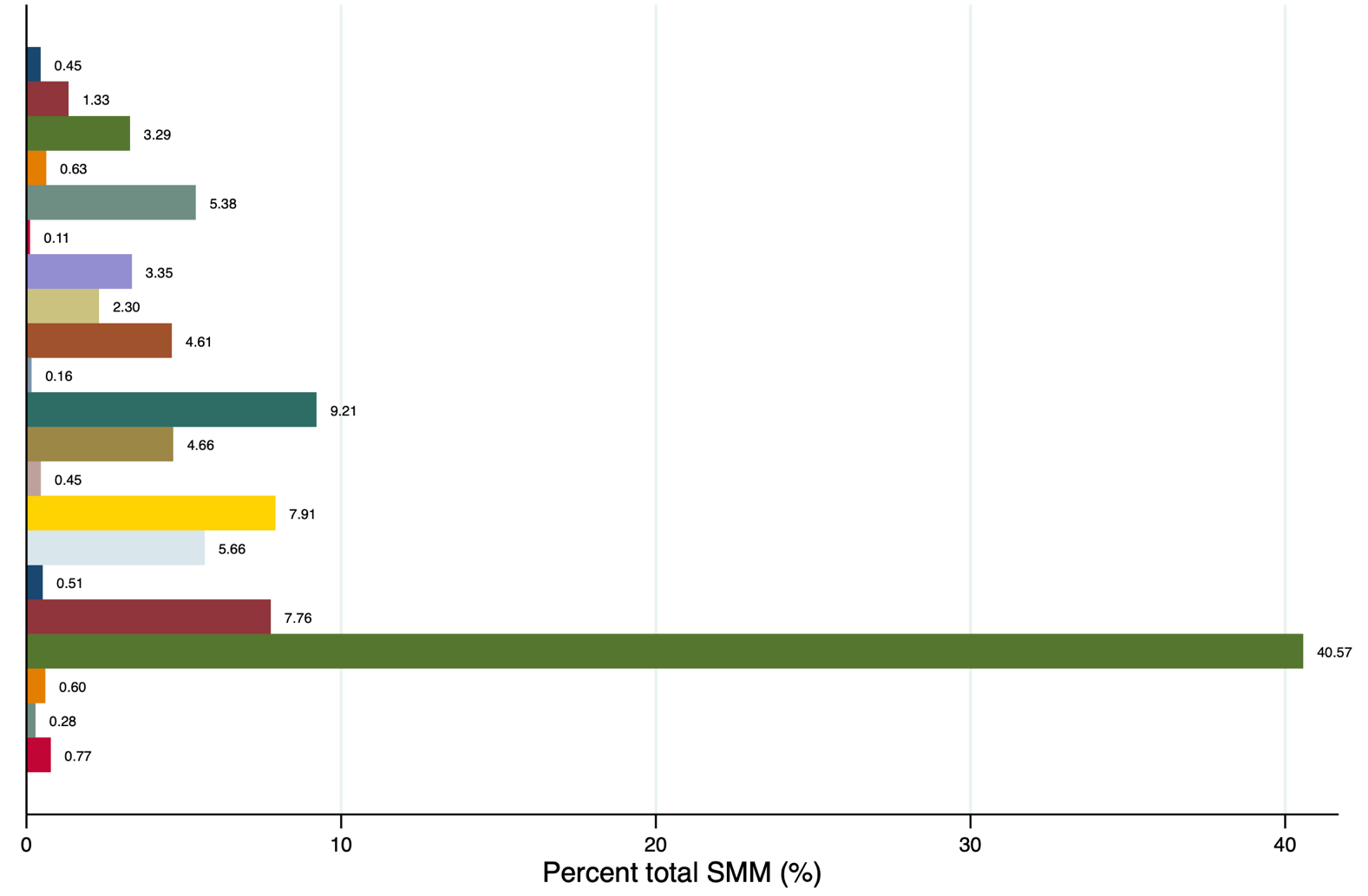
**

**Table S7.** Spearman’s and biserial correlation coefficients between the different measures of disadvantage. Red=strong correlation, Orange = moderate correlation, Green = weak correlation

|  | **SES-WOA Employment** | **SES-WOA**  **Welfare** | **SES-WOA**  **Education** | **Liveability** | **Household Disposable Income** | **Educational Attainment** | **Employment** | **SES-WOA**  **Composite** |
| --- | --- | --- | --- | --- | --- | --- | --- | --- |
| **SES-WOA Employment** | 1 |  |  |  |  |  |  |  |
| **SES-WOA**  **Welfare** | 0.91 | 1.000 |  |  |  |  |  |  |
| **SES-WOA**  **Education** | 0.65 | 0.73 | 1.000 |  |  |  |  |  |
| **Liveability** | 0.44 | 0.53 | 0.50 | 1.00 |  |  |  |  |
| **Household Disposable Income** | 0.28 | 0.33 | 0.37 | 0.25 | 1.00 |  |  |  |
| **Educational attainment** | 0.18 | 0.23 | 0.34 | 0.22 | 0.52 | 1.00 |  |  |
| **Individual**  **Employment** | 0.18 | 0.17 | 0.16 | 0.13 | 0.38 | 0.29 | 1.00 |  |
| **SES-WOA**  **Composite** | 0.93 | 0.98 | 0.83 | 0.52 | 0.35 | 0.26 | 0.18 | 1.00 |

**Table S8.** Cross-tabulation of the individual measures of socioeconomic disadvantage N (%)

|  | **Individual Educational Attainment** | | | **Individual Employment** | |
| --- | --- | --- | --- | --- | --- |
|  | **Low** | **Medium** | **High** | **Unemployed** | **Employed** |
|  | **N=95,845** | **N=270,252** | **N=285,074** | **N=200,699** | **N=632,167** |
| **Household disposable income (euros)** |  |  |  |  |  |
| <€20,000 | 41,673 (44.1) | 37,219 (13.8) | 9,159  (3.2) | 75,370  (43.1) | 35,720  (6.3) |
| €20-39,999 | 19,777 (20.9) | 41,626 (15.5) | 14,282  (5.0) | 34,503  (19.7) | 55,823  (9.9) |
| €40-59,999 | 15,852 (16.8) | 65,330 (24.3) | 34,544  (12.2) | 25,126  (14.4) | 107,882  (19.1) |
| €60-79,999 | 11,248 (11.9) | 77,498 (28.8) | 79,248  (27.9) | 18,763  (10.7) | 167,420  (29.6) |
| €80-100,00 | 5,980  (6.3) | 47,627 (17.7) | 147,120 (51.7) | 21,323  (12.2) | 199,018  (35.2) |
| **Individual educational attainment** |  |  |  |  |  |
| Low |  |  |  | 50,600 (36.9) | 45,245  (8.8) |
| Medium |  |  |  | 54,047  (39.4) | 216,205  (42.1) |
| High |  |  |  | 32,641  (23.8) | 252,433  (49.1) |

**Table** **S9.** Univariable and multivariable analysis showing risk ratios (95% CI) between **SES-WOA** and SMM compared to the least SES-WOA disadvantaged quintile. *Model 1: Adjusted for year, age, parity, migration status and ethnicity **Model 2: Model 1 + additionally adjusted for pre-existing medical conditions, substance misuse, and pre-existing mental health conditions ***Model 3: Model 1 + additionally adjusted for neighborhood livability. ****Model 4: Model 1 + additionally adjusted for individual measures of SES (household income, educational attainment and employment status) ICC = Intra-class Correlation Coefficient

| **SES-WOA** | **Univariable** | **Model 1*** | **Model 2**** | **Model 3***** | **Model 4***** |
| --- | --- | --- | --- | --- | --- |
| Most disadvantaged 20% | 1.31  (1.23-1.40) | 1.23  (1.15-1.31) | 1.20  (1.11-1.28) | 1.23  (1.15-1.33) | 1.12  (1.04-1.20) |
| More disadvantaged 20-40% | 1.12  (1.05-1.19) | 1.11  (1.04-1.19) | 1.09  (1.02-1.17) | 1.11  (1.03-1.19) | 1.04  (0.97-1.12) |
| Less disadvantaged 40-60% | 1.11  (1.03-1.19) | 1.11  (1.03-1.19) | 1.10  (1.03-1.18) | 1.13  (1.05-1.21) | 1.07  (0.99-1.15) |
| Less disadvantaged 60-80% | 1.06  (0.99-1.14) | 1.08  (1.00-1.16) | 1.07  (1.00-1.15) | 1.08  (1.00-1.16) | 1.05  (0.98-1.14) |
| Least disadvantaged 80-100% | 1(ref) | 1(ref) | 1(ref) | 1(ref) | 1(ref) |
| ICC | 0.014  (0.011-0.018) | 0.015  (0.010-0.018) | 0.015  (0.011- 0.019) | 0.013  (0.010-0.018) | 0.015  (0.011-0.020) |

**Table S10.** Univariable and multivariable analysis showing risk ratios (95% CI) between **SES-WOA Education** and severe maternal morbidity compared to the least SES-WOA Education disadvantaged quintile. *Model 1: Adjusted for year, age, parity, migration status and ethnicity **Model 2: Model 1 + additionally adjusted for pre-existing medical conditions, substance misuse, and pre-existing mental health conditions ***Model 3: Model 1 + additionally adjusted for individual educational attainment. ****Model 4: Model 1 + additionally adjusted for individual measures of socioeconomic disadvantage (household income, educational attainment and employment status) ICC = Intra-class Correlation Coefficient

| **SES-WOA Education** | **Univariable** | **Model 1*** | **Model 2**** | **Model 3***** | **Model 4****** |
| --- | --- | --- | --- | --- | --- |
| Most disadvantaged 20% | 1.27  (1.20-1.34) | 1.21  (1.14-1.28) | 1.16  (1.09-1.23) | 1.12  (1.05-1.19) | 1.09  (1.01-1.16) |
| More disadvantaged 20-40% | 1.14  (1.08-1.21) | 1.13  (1.07-1.20) | 1.10  (1.03-1.16) | 1.08  (1.01-1.15) | 1.06  (1.00-1.14) |
| Less disadvantaged 40-60% | 1.12  (1.05-1.19) | 1.12  (1.05-1.19) | 1.09  (1.02-1.16) | 1.08  (1.01-1.16) | 1.07  (1.00-1.15) |
| Less disadvantaged 60-80% | 1.03  (1.00-1.01) | 1.03  (0.97-1.10) | 1.01  (0.95-1.08) | 1.00  (0.93-1.01) | 1.00  (0.93-1.07) |
| Least disadvantaged 80-100% | 1(ref) | 1(ref) | 1(ref) | 1(ref) | 1(ref) |
| ICC | 0.015  (0.012-0.020) | 0.014  (0.010-0.018) | 0.015  (0.011-0.019) | 0.015  (0.011- 0.020) | 0.015  (0.011-0.020) |

**Table S11.** Univariable and multivariable analysis showing risk ratios (95% CI) between **SES-WOA Employment** and severe maternal morbidity compared to the least SES-WOA Employment disadvantaged quintile. *Model 1: Adjusted for year, age, parity, migration status and ethnicity **Model 2: Model 1 + additionally adjusted for pre-existing medical conditions, substance misuse, and pre-existing mental health conditions ***Model 3: Model 1 + additionally adjusted for individual employment status. ****Model 4: Model 1 + additionally adjusted for individual measures of socioeconomic disadvantage (household income-educational attainment and employment status) ICC = Intra-class Correlation Coefficient

| **SES-WOA Employment** | **Univariable** | **Model 1*** | **Model 2**** | **Model 3***** | **Model 4****** |
| --- | --- | --- | --- | --- | --- |
| Most disadvantaged 20% | 1.26  (1.19-1.33) | 1.17  (1.10-1.24) | 1.13  (1.06-1.20) | 1.14  (1.07-1.21) | 1.08  (1.01-1.15) |
| More disadvantaged 20-40% | 1.07  (1.01-1.14) | 1.06  (0.99-1.13) | 1.05  (0.98-1.11) | 1.05  (0.98-1.12) | 1.01  (0.94-1.08) |
| Less disadvantaged 40-60% | 1.06  (1.00-1.13) | 1.06  (1.00-1.13) | 1.06  (1.00-1.13) | 1.06  (1.00-1.13) | 1.01  (0.95-1.09) |
| Less disadvantaged 60-80% | 1.01  (0.94-1.08) | 1.01  (0.95-1.09) | 1.01  (0.94-1.08) | 1.01  (0.94-1.08) | 1.01  (0.93-1.08) |
| Least disadvantaged 80-100% | 1(ref) | 1(ref) | 1(ref) | 1(ref) | 1(ref) |
| ICC | 0.014  (0.011-0.018) | 0.014  (0.010-0.018) | 0.014  (0.010- 0.018) | 0.015  (0.011-0.019) | 0.014  (0.011-0.020) |

**Table S12.** Univariable and multivariable analysis showing risk ratios (95% CI) between **SES-WOA Welfare** and severe maternal morbidity compared to the least SES-WOA Welfare disadvantaged quintile. *Model 1: Adjusted for year, age, parity, migration status and ethnicity **Model 2: Model 1 + additionally adjusted for pre-existing medical conditions, substance misuse, and pre-existing mental health conditions ***Model 3: Model 1 + additionally adjusted for household disposable income. ****Model 4: Model 1 + additionally adjusted for individual measures of socioeconomic disadvantage (household income-educational attainment and employment status) ICC = Intra-class Correlation Coefficient

| **SES-WOA Welfare** | **Univariable** | **Model 1*** | **Model 2**** | **Model 3***** | **Model 4****** |
| --- | --- | --- | --- | --- | --- |
| Most disadvantaged 20% | 1.32  (1.23-1.42) | 1.24  (1.15-1.34) | 1.17  (1.08-1.27) | 1.21  (1.11-1.29) | 1.14  (1.05-1.24) |
| More disadvantaged 20-40% | 1.13  (1.05-1.22) | 1.12  (1.03-1.21) | 1.08  (0.99-1.17) | 1.10  (1.01-1.81) | 1.07  (0.98-1.17) |
| Less disadvantaged 40-60% | 1.14  (1.05-1.23) | 1.14  (1.05-1.23) | 1.12  (1.04-1.22) | 1.13  (1.04-1.22) | 1.13  (1.03-1.22) |
| Less disadvantaged 60-80% | 1.07  (0.99-1.15) | 1.07  (0.99-1.16) | 1.05  (0.97-1.15) | 1.06  (0.98-1.15) | 1.07  (0.97-1.16) |
| Least disadvantaged 80-100% | 1(ref) | 1(ref) | 1(ref) | 1(ref) | 1(ref) |
| ICC | 0.014  (0.011-0.019) | 0.014  (0.010-0.018) | 0.013  (0.010-0.017) | 0.015  (0.011-0.019) | 0.015  (0.011-0.020) |

**Table S13.** Univariable and multivariable analysis showing risk ratios (95% CI) between neighbourhood **Liveability** and severe maternal morbidity compared to the least neighbourhood disadvantage category. *Model 1: Adjusted for year, age, parity, migration status and ethnicity **Model 2: Model 1 + additionally adjusted for pre-existing medical conditions-substance misuse, and pre-existing mental health conditions ***Model 3: Model 1 + additionally adjusted for SES-WOA. ****Model 4: Model 1 + additionally adjusted for individual measures of socioeconomic disadvantage (household income-educational attainment and employment status) ICC = Intra-class Correlation Coefficient

| **Liveability** | **Univariable** | **Model 1*** | **Model 2**** | **Model 3***** | **Model 4****** |
| --- | --- | --- | --- | --- | --- |
| Lowest | 1.26  (1.16-1.36) | 1.13  (1.04-1.23) | 1.09  (1.00-1.19) | 1.00  (0.91-1.10) | 1.04  (0.95-1.14) |
| Lower | 1.20  (1.11-1.29) | 1.13  (1.04-1.23) | 1.09  (1.01-1.18) | 1.02  (0.94-1.11) | 1.05  (0.97-1.15) |
| Middle | 1.06  (1.00-1.12) | 1.04  (0.98-1.10) | 1.01  (0.95-1.08) | 0.98  (0.92-1.04) | 1.01  (0.94-1.07) |
| Higher | 1.02  (0.96-1.08) | 1.01  (0.95-1.08) | 1.00  (0.94-1.06) | 0.98  (0.92-1.05) | 1.00  (0.93-1.07) |
| Highest | 1(ref) | 1(ref) | 1(ref) | 1(ref) | 1(ref) |
| ICC | 0.016  (0.012-0.021) | 0.015  (0.011-0.020) | 0.016  (0.012-0.021) | 0.016  (0.012-0.020) | 0.016  (0.012-0.020) |

**Table S14.** Univariable and multivariable analysis showing risk ratios (95% CI) between **Household Disposable Income** and severe maternal morbidity compared to the highest Household Disposable Income quintile. *Model 1: Adjusted for year, age, parity, migration status and ethnicity **Model 2: Model 1 + additionally adjusted for pre-existing medical conditions, substance misuse, and pre-existing mental health conditions ***Model 3: Model 1 + additionally adjusted for SES-WOA Welfare (multilevel model). ICC = Intra-class Correlation Coefficient

| **Household Disposable Income (euros)** | **Univariable** | **Model 1*** | **Model 2**** | **Model 3***** |
| --- | --- | --- | --- | --- |
| <€20,000 | 1.40  (1.33-1.48) | 1.33  (1.24-1.41) | 1.27  (1.19-1.35) | 1.29  (1.21-1.37) |
| €20-39,999 | 1.31  (1.24-1.39) | 1.30  (1.22-1.38) | 1.23  (1.15-1.30) | 1.26  (1.18-1.35) |
| €40-59,999 | 1.17  (1.11-1.23) | 1.19  (1.12-1.26) | 1.15  (1.08-1.21) | 1.17  (1.10-1.24) |
| €60-79,999 | 1.05  (1.00-1.11) | 1.07  (1.02-1.13) | 1.05  (1.00-1.11) | 1.06  (1.01-1.12) |
| €80-100,00 | 1(ref) | 1(ref) | 1(ref) | 1(ref) |
| ICC |  |  |  | 0.015  (0.011-0.019) |

**Table S15.** Univariable and multivariable analysis showing risk ratios (95% CI) between **Unemployment** and severe maternal morbidity compared to employed women. *Model 1: Adjusted for year, age, parity, migration status and ethnicity **Model 2: Model 1 + additionally adjusted for pre-existing medical conditions, substance misuse,and pre-existing mental health conditions ***Model 3: Model 1 + additionally adjusted for SES-WOA Employment (multilevel model). ICC = Intra-class Correlation Coefficient

| **Employment Status** | **Univariable** | **Model 1*** | **Model 2**** | **Model 3***** |
| --- | --- | --- | --- | --- |
| Unemployed | 1.32  (1.28-1.37) | 1.25  (1.20-1.30) | 1.22  (1.17-1.27) | 1.23  (1.18-1.28) |
| Employed | 1(ref) | 1(ref) | 1(ref) | 1(ref) |
| ICC |  |  |  | 0.015  (0.011-0.019) |

**Table S16.** Univariable and multivariable analysis showing risk ratios (95% CI) between **Individual Educational Attainment** and severe maternal morbidity compared to women with the highest educational attainment. *Model 1: Adjusted for year, age, parity, migration status and ethnicity **Model 2: Model 1 + additionally adjusted for pre-existing medical conditions, substance misuse, and pre-existing mental health conditions ***Model 3: Model 1 + additionally adjusted for SES-WOA Education (multilevel model). ICC = Intra-class Correlation Coefficient

|  | **Univariable** | **Model 1*** | **Model 2**** | **Model 3***** |
| --- | --- | --- | --- | --- |
| Low | 1.50  (1.43-1.59) | 1.41  (1.33-1.50) | 1.29  (1.22-1.37) | 1.38  (1.30-1.46) |
| Medium | 1.29  (1.24-1.35) | 1.29  (1.23-1.34) | 1.20  (1.15-1.25) | 1.26  (1.20-1.32) |
| High | 1(ref) | 1(ref) | 1(ref) | 1(ref) |
| ICC |  |  |  | 0.015  (0.011- 0.020) |

**Figure S5.** The risk ratios and their 95% confidence intervals (RR (95% CI)) of each condition comprising the high proportion of severe maternal morbidity (SMM) in each SES-WOA quintile compared to the least disadvantaged SES-WOA quintile (Model 1). Least= Least disadvantaged 80-100%, Most = Most disadvantaged 20%


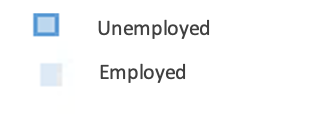

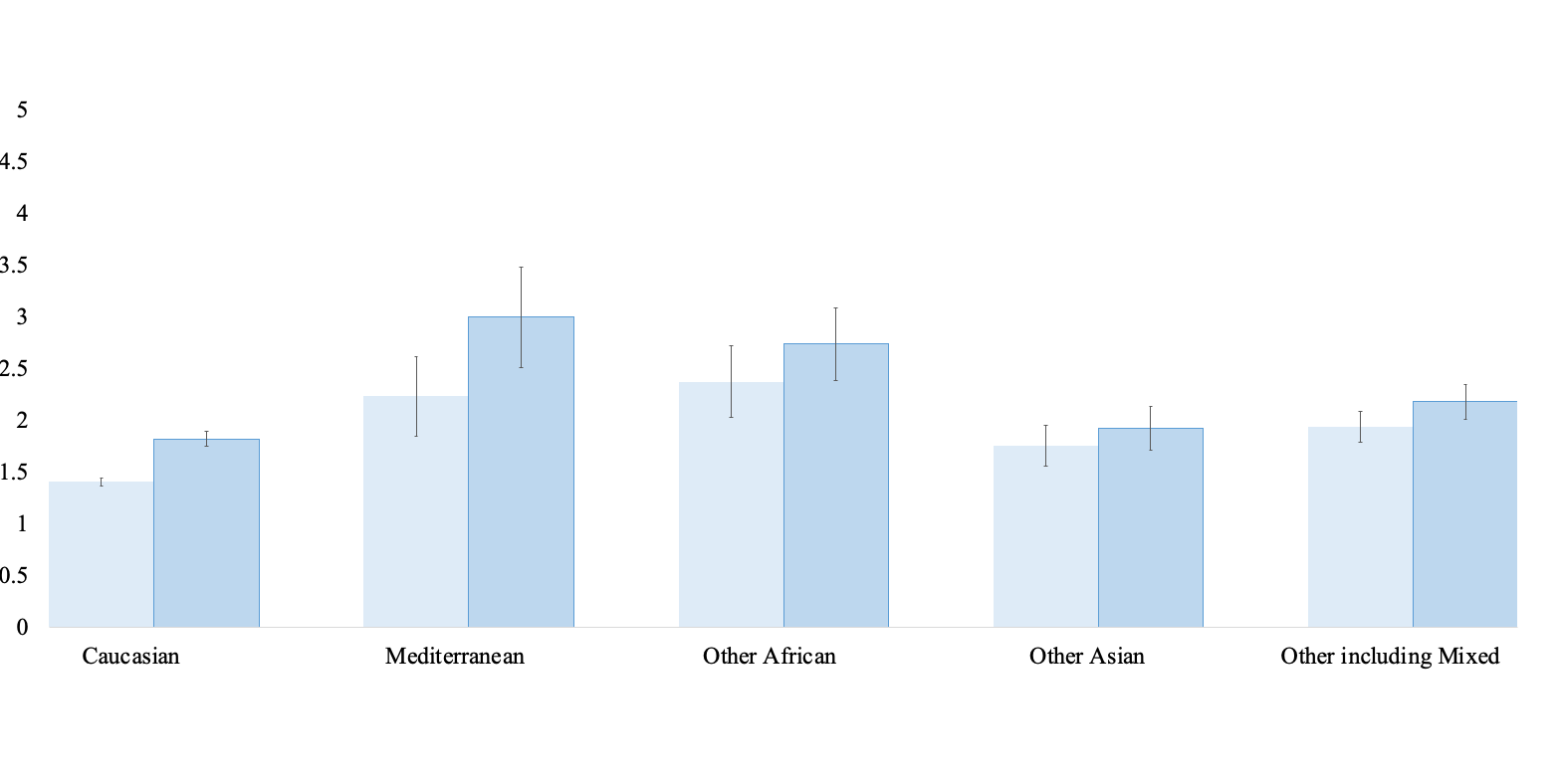


P_interaction_ = 0.02

Predictive margins and 95% CI (%)

**Figure S6.** The average adjusted predictive margins (%) and their 95% confidence intervals (95% CI) of severe maternal morbidity for Unemployed and Employed women stratified by ethnicity


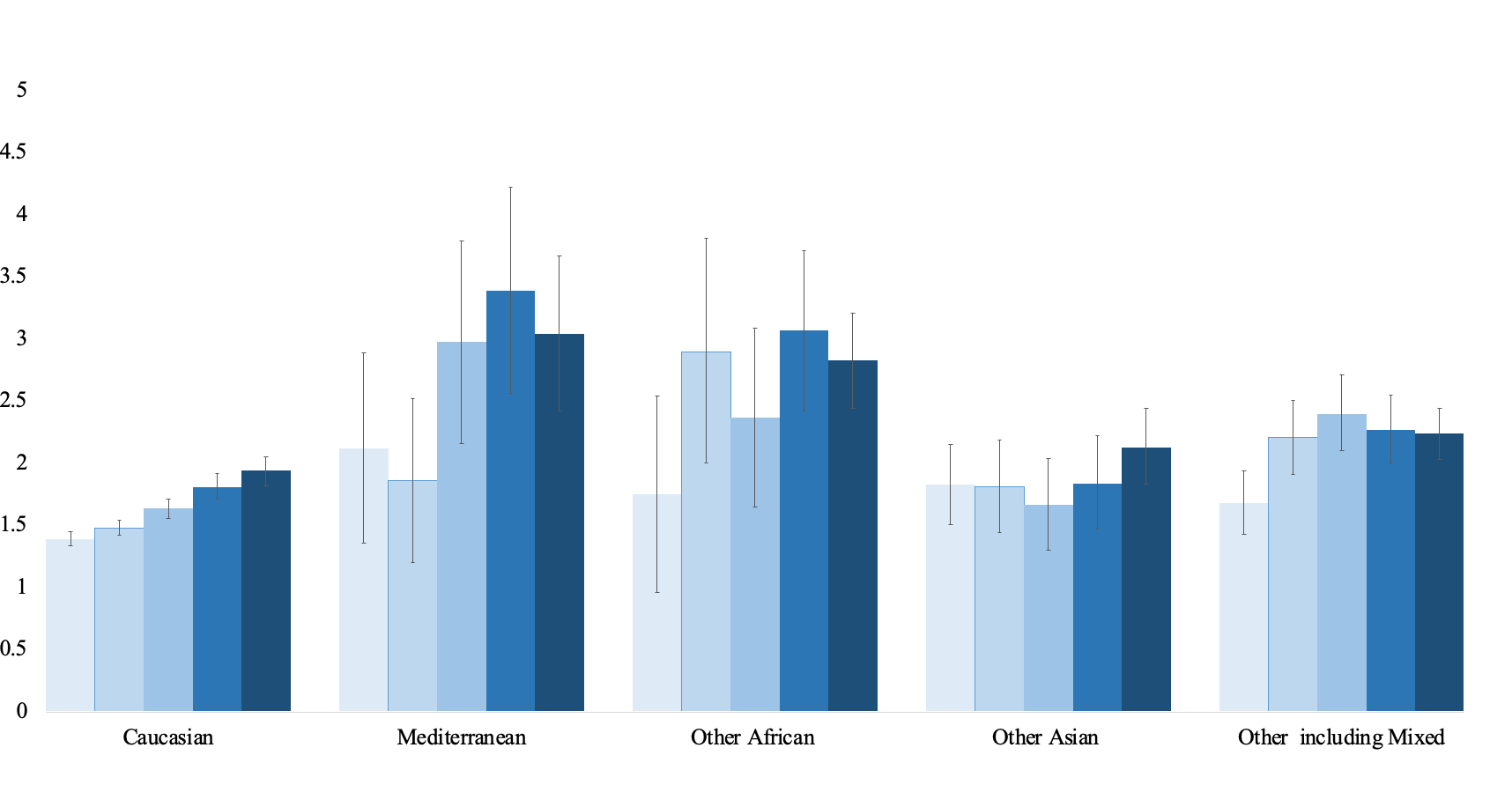

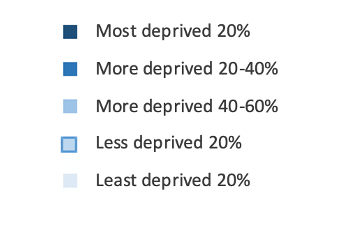

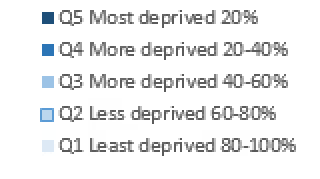


Predictive margins and 95% CI (%)

P_interaction_ = 0.03

**Figure S7.** The average adjusted predictive margins (%) and their 95% confidence intervals (95% CI) of severe maternal morbidity in each Household Disposable Income quintile stratified by ethnicity


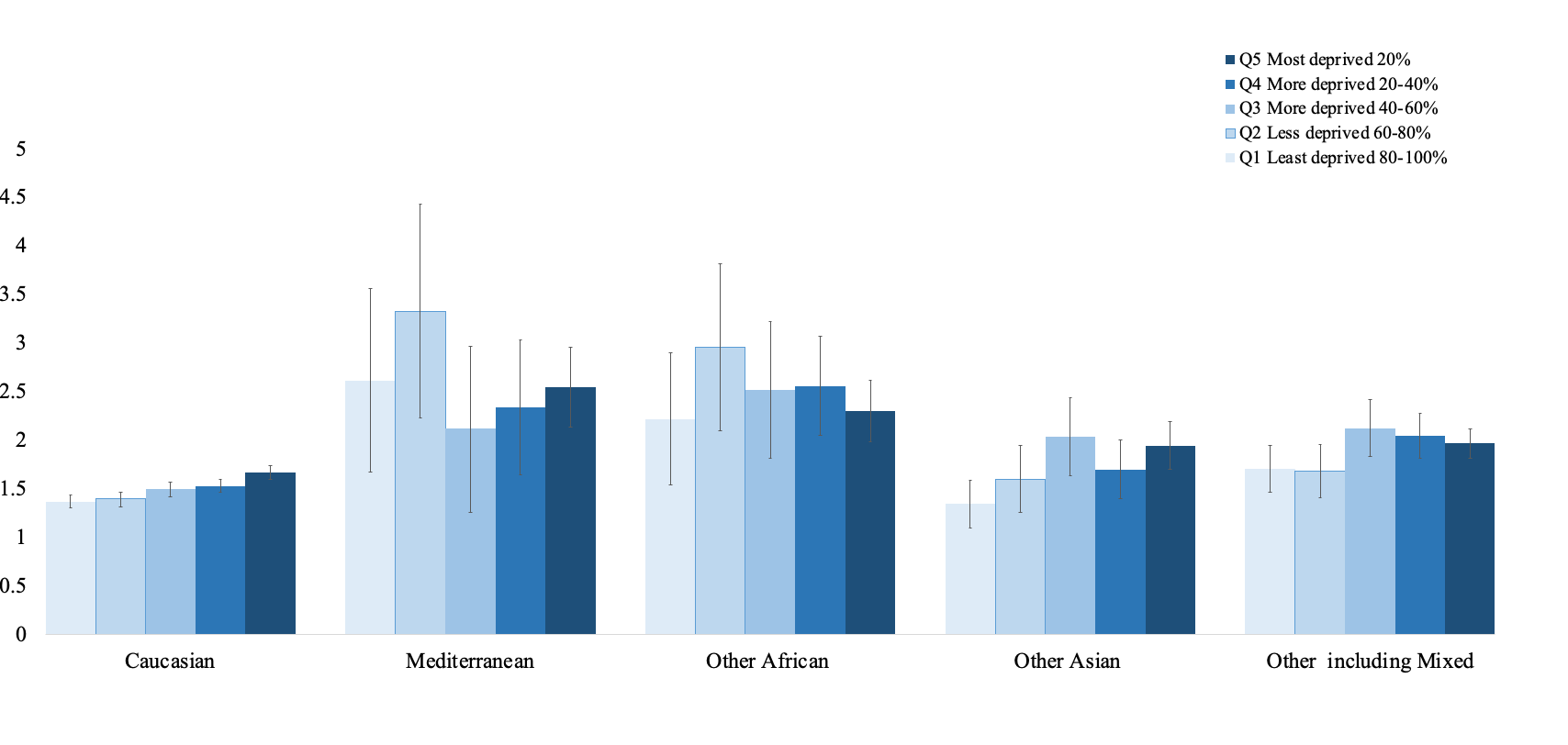

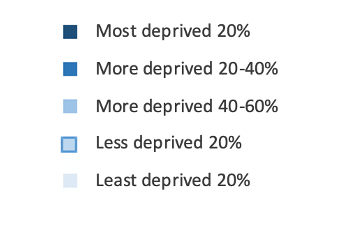


P_interaction_ = 0.045

**Figure S8.** The average adjusted predictive margins (%) and their 95% confidence intervals (95% CI) of severe maternal morbidity in each SES-WOA Education quintile stratified by ethnicity

Predictive margins and 95% CI (%)

**Table S17.** Number of severe maternal morbidity (SMM) cases, total, rate of SMM, and adjusted risk ratios (aRR) and their 95% confidence intervals (95% CI) of SMM for Unemployed women compared to Caucasian Employed women stratified by ethnic group and adjusted for age, parity, year and migration status.

| **Employment status** | **SMM N** | **Total N** | **Rate** | **aRR (95% CI)** |
| --- | --- | --- | --- | --- |
| **Caucasian** | | | | |
| Employed | 7,676 | 539,282 | 1.42 | 1(ref) |
| Unemployed | 2,209 | 120,002 | 1.84 | 1.30(1.24-1.37) |
| **Mediterranean ((North African + Turkish)** | | | | |
| Employed | 138 | 6,763 | 2.04 | 1.59 (1.33-1.90) |
| Unemployed | 158 | 5,758 | 2.74 | 2.13 (1.81-2.52) |
| **Other African** | | | | |
| Employed | 198 | 8,724 | 2.27 | 1.69 (1.46-1.96) |
| Unemployed | 259 | 10,053 | 2.58 | 1.95 (1.71-2.22) |
| **Other Asian** | | | | |
| Employed | 337 | 20,088 | 1.68 | 1.25 (1.11-1.40) |
| Unemployed | 338 | 18,561 | 1.82 | 1.37 (1.22-1.54) |
| **Other including Mixed** | | | | |
| Employed | 781 | 41,919 | 1.86 | 1.38 (1.27-1.50) |
| Unemployed | 849 | 40,954 | 2.07 | 1.55 (1.43-1.69) |

**Table S18.** Number of severe maternal morbidity (SMM) cases, total in each quintile, rate of SMM, and adjusted risk ratios (aRR) and their 95% confidence intervals (95% CI) of SMM for each Household Disposable Income quintile and SMM, compared to the Caucasian women with a Household Disposable Income €80,000-€100,000, stratified by ethnic group adjusted for age, parity, year and migration status.

| **Household Disposable Income (euros)** | **SMM N** | **Total N** | **Rate (%)** | **aRR (95% CI)** |
| --- | --- | --- | --- | --- |
| **Caucasian** | | | | |
| €80-100,00 | 2,793 | 194,001 | 1.44 | 1 (ref) |
| €60-79,999 | 2,429 | 162,811 | 1.49 | 1.06 (1.00 1.12) |
| €40-59,999 | 1,785 | 109,497 | 1.63 | 1.18 (1.11 1.25) |
| €20-39,999 | 1,157 | 63,881 | 1.81 | 1.30 (1.21 1.40) |
| <€20,000 | 1,136 | 58,699 | 1.94 | 1.39 (1.29 1.50) |
| **Mediterranean ((North African + Turkish)** | | | | |
| €80-100,00 | 30 | 1,547 | 1.94 | 1.52 (1.06 2.19) |
| €60-79,999 | 31 | 1,850 | 1.68 | 1.34 (0.93 1.91) |
| €40-59,999 | 53 | 1,977 | 2.68 | 2.14 (1.62 2.82) |
| €20-39,999 | 66 | 2,162 | 3.05 | 2.43 (1.89 3.12) |
| <€20,000 | 96 | 3,505 | 2.74 | 2.18 (1.77 2.70) |
| **Other African** | | | | |
| €80-100,00 | 19 | 1,120 | 1.70 | 1.26 (0.80 1.98) |
| €60-79,999 | 40 | 1,434 | 2.79 | 2.09 (1.52 2.86) |
| €40-59,999 | 42 | 1,869 | 2.25 | 1.70 (1.25 2.32) |
| €20-39,999 | 90 | 3,115 | 2.89 | 2.20 (1.77 2.73) |
| <€20,000 | 230 | 8,825 | 2.61 | 2.03 (1.76 2.35) |
| **Other Asian** | | | | |
| €80-100,00 | 133 | 7,515 | 1.77 | 1.31 (1.10 1.57) |
| €60-79,999 | 92 | 5,321 | 1.73 | 1.30 (1.05 1.61) |
| €40-59,999 | 81 | 5,108 | 1.59 | 1.20 (0.96 1.50) |
| €20-39,999 | 95 | 5,475 | 1.74 | 1.32 (1.07 1.63) |
| <€20,000 | 204 | 10,291 | 1.98 | 1.53 (1.32 1.78) |
| **Other including Mixed** | | | | |
| €80-100,00 | 176 | 10,733 | 1.64 | 1.21 (1.03 1.41) |
| €60-79,999 | 220 | 10,392 | 2.12 | 1.59 (1.38 1.83) |
| €40-59,999 | 259 | 11,399 | 2.27 | 1.73 (1.51 1.98) |
| €20-39,999 | 288 | 13,418 | 2.15 | 1.63 (1.43 1.86) |
| <€20,000 | 557 | 26,642 | 2.09 | 1.61 (1.45 1.78) |

**Table S19.** Number of severe maternal morbidity (SMM) cases, total in each quintile, rate of SMM and adjusted risk ratios (aRR) and their 95% confidence intervals (95% CI) of SMM for each SES-WOA Education quintile compared to Caucasian women living in the least disadvantaged SES-WOA Education quintile stratified by ethnic group and adjusted for age, parity, year and migration status.

| **SESWOA Education Quintile** | **SMM N** | **Total N** | **Rate %** | **aRR (95% CI)** |
| --- | --- | --- | --- | --- |
| **Caucasian** | | | | |
| Least disadvantaged 20% | 1,848 | 133,785 | 1.38 | 1(ref) |
| Less disadvantaged 20-40% | 1,503 | 108,005 | 1.39 | 1.01 (0.95-1.09) |
| Less disadvantage 40-60% | 1,774 | 119,752 | 1.48 | 1.09 (1.02-1.16) |
| More disadvantaged 60-80% | 2,253 | 148,578 | 1.52 | 1.12 (1.05-1.19) |
| Most disadvantaged 80-100% | 2,507 | 149,164 | 1.68 | 1.23 (1.15-1.31) |
| **Mediterranean ((North African + Turkish)** | | | | |
| Least disadvantaged 20% | 30 | 1,229 | 2.44 | 1.91 (1.32-2.75) |
| Less disadvantaged 20-40% | 36 | 1,161 | 3.10 | 2.44 (1.75-3.42) |
| Less disadvantage 40-60% | 24 | 1,236 | 1.94 | 1.55 (1.03-2.33) |
| More disadvantaged 60-80% | 45 | 2,088 | 2.16 | 1.75 (1.29-2.36) |
| Most disadvantaged 80-100% | 161 | 6,807 | 2.37 | 1.88 (1.58-2.23) |
| **Other African** | | | | |
| Least disadvantaged 20% | 41 | 1,808 | 2.27 | 1.69 (1.23-2.31) |
| Less disadvantaged 20-40% | 46 | 1,537 | 2.99 | 2.25 (1.67-3.03) |
| Less disadvantage 40-60% | 50 | 1,986 | 2.52 | 1.92 (1.44-2.56) |
| More disadvantaged 60-80% | 98 | 3,863 | 2.54 | 1.96 (1.59-2.42) |
| Most disadvantaged 80-100% | 222 | 9,583 | 2.32 | 1.73 (1.49-2.01) |
| **Other Asian** | | | | |
| Least disadvantaged 20% | 116 | 8,384 | 1.38 | 1.03 (0.85-1.25) |
| Less disadvantaged 20-40% | 84 | 5,167 | 1.63 | 1.22 (0.98-1.53) |
| Less disadvantage 40-60% | 102 | 4,972 | 2.05 | 1.56 (1.27-1.92) |
| More disadvantaged 60-80% | 123 | 7,228 | 1.70 | 1.31 (1.08-1.58) |
| Most disadvantaged 80-100% | 250 | 12,898 | 1.94 | 1.48 (1.28-1.70) |
| **Other including Mixed** | | | | |
| Least disadvantaged 20% | 204 | 11,487 | 1.78 | 1.31 (1.13-1.52) |
| Less disadvantaged 20-40% | 146 | 8,522 | 1.71 | 1.28 (1.08-1.53) |
| Less disadvantage 40-60% | 211 | 9,826 | 2.15 | 1.62 (1.39-1.88) |
| More disadvantaged 60-80% | 317 | 15,381 | 2.06 | 1.58 (1.39-1.80) |
| Most disadvantaged 80-100% | 752 | 37,657 | 2.00 | 1.51 (1.37-1.67) |

**Figure S9.** The average adjusted predictive margins (%) and their 95% confidence intervals (95% CI) of severe maternal morbidity for Unemployed and Employed women stratified by ethnicity and migration status


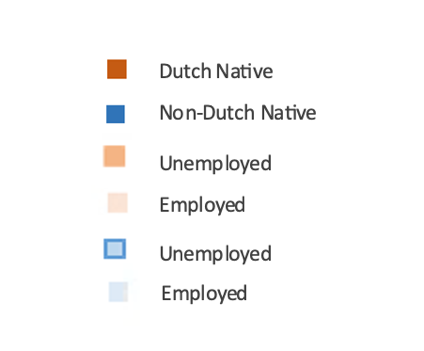


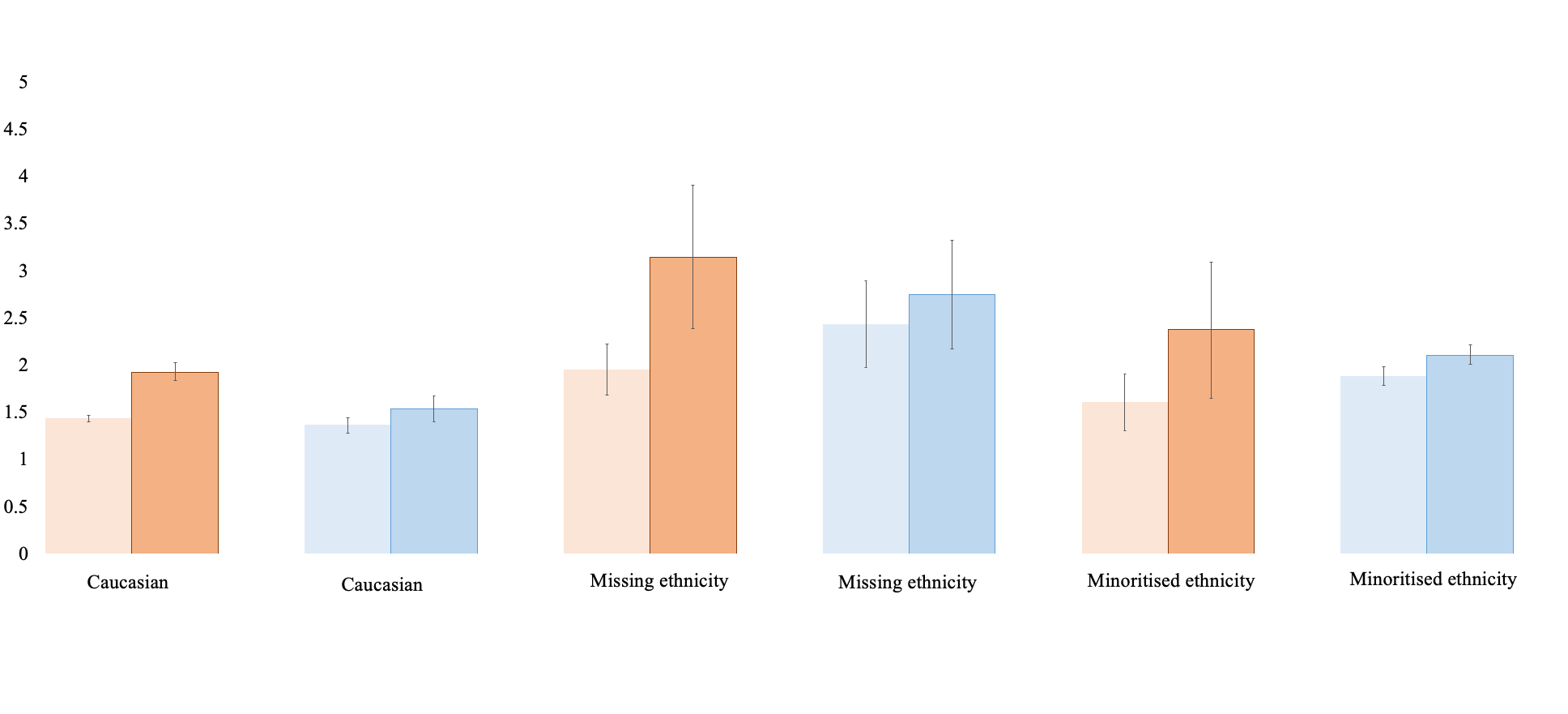


Predictive margins and 95% CI (%)

P_interaction_ <0.001


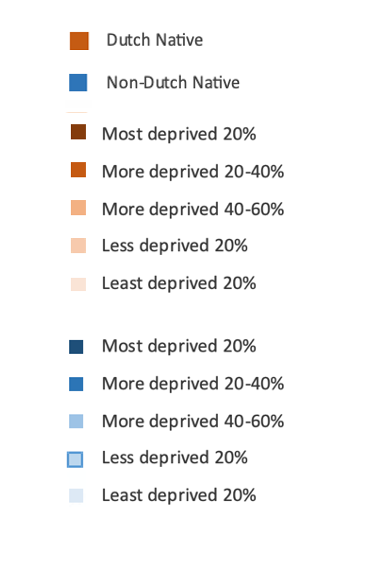

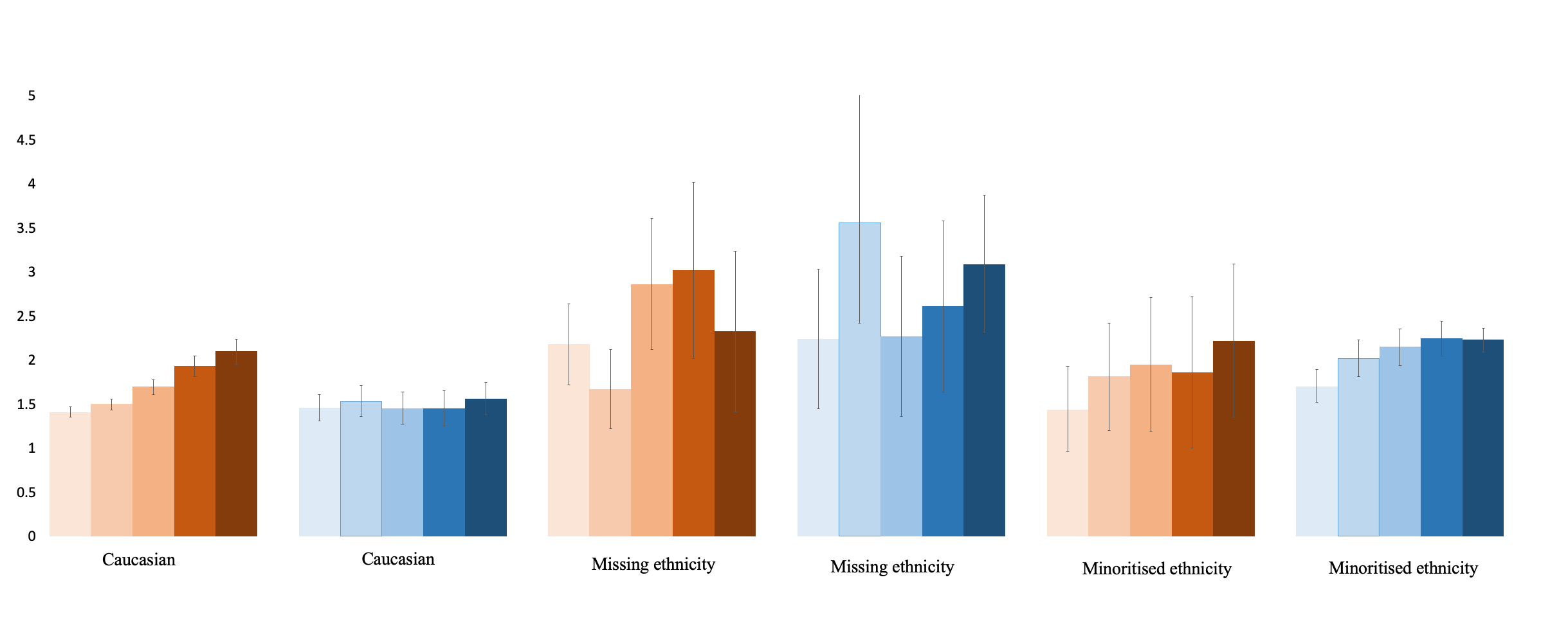


Predictive margins and 95% CI (%)

P_interaction_ <0.001

**Figure S10.** The average adjusted predictive margins (%) and their 95% confidence intervals (95% CI) of severe maternal morbidity in each Household Disposable Income quintile stratified by ethnicity and migration status


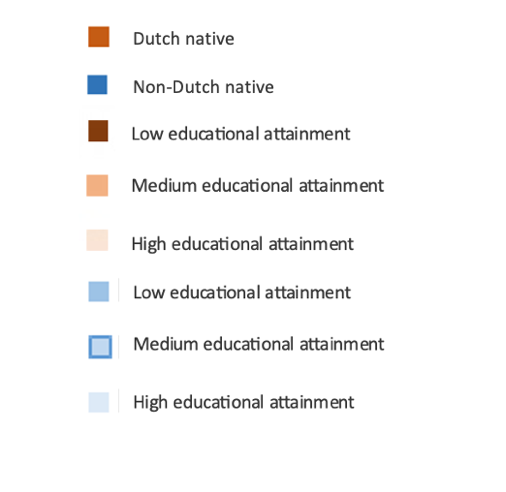

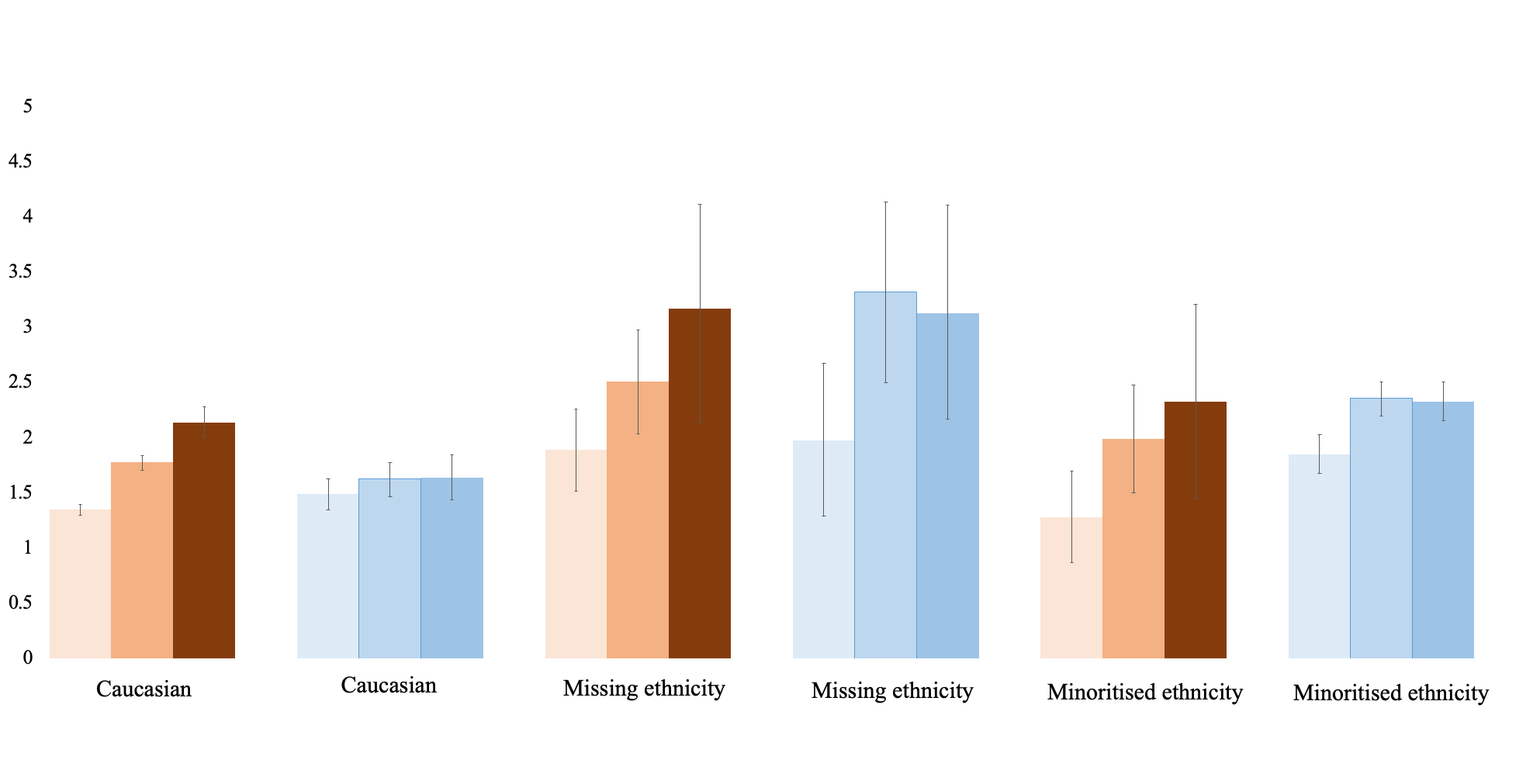


Predictive margins and 95% CI (%)

P_interaction_ <0.001

**Figure S11.** The average adjusted predictive margins (%) and their 95% confidence intervals (95% CI) of severe maternal morbidity at each level of Educational Attainment stratified by ethnicity and migration status

**Table S20**. Number of severe maternal morbidity (SMM) cases, total in each quintile, rate of SMM and adjusted risk ratios (aRR) and their 95% confidence intervals (95% CI) for Household Disposable Income quintile and SMM, compared to Caucasian women with a Household Disposable Income €80,000-€100,000 stratified by ethnicity and migration status and adjusted for age, parity and year.

| **Household Disposable Income Quintile (euros)** | **SMM N** | **Total N** | **Rate %** | **aRR (95% CI)** |
| --- | --- | --- | --- | --- |
| **Caucasian and Dutch native** | |  |  |  |
| €80-100,00 | 2,438 | 170,405 | 1.43 | 1 (ref) |
| €60-79,999 | 2,140 | 144,122 | 1.48 | 1.06 (1.00-1.13) |
| €40-59,999 | 1,550 | 93,322 | 1.66 | 1.20 (1.13-1.29) |
| €20-39,999 | 959 | 50,277 | 1.91 | 1.37 (1.27-1.48) |
| <€20,000 | 856 | 40,863 | 2.09 | 1.49 (1.37-1.61) |
| **Caucasian and non-Dutch native** | | |  |  |
| €80-100,00 | 355 | 23,596 | 1.50 | 1.04 (0.93-1.16) |
| €60-79,999 | 289 | 18,689 | 1.55 | 1.09 (0.96-1.23) |
| €40-59,999 | 235 | 16,175 | 1.45 | 1.03 (0.90-1.18) |
| €20-39,999 | 198 | 13,604 | 1.46 | 1.03 (0.89-1.19) |
| <€20,000 | 280 | 17,836 | 1.57 | 1.11 (0.98-1.26) |
| **Minoritised ethnicity and Dutch native** | | |  |  |
| €80-100,00 | 30 | 1,535 | 1.95 | 1.02 (0.73-1.44) |
| €60-79,999 | 31 | 1,836 | 1.69 | 1.29 (0.92-1.81) |
| €40-59,999 | 53 | 1,962 | 2.70 | 1.38 (0.93-2.05) |
| €20-39,999 | 66 | 2,141 | 3.08 | 1.32 (0.83-2.10) |
| <€20,000 | 96 | 3,479 | 2.76 | 1.57 (1.06-2.34) |
| **Other including Mixed and non-Dutch native** | | | |  |
| €80-100,00 | 18 | 1,067 | 1.69 | 1.20 (1.03-1.38) |
| €60-79,999 | 39 | 1,378 | 2.83 | 1.50 (1.31-1.72) |
| €40-59,999 | 41 | 1,802 | 2.28 | 1.59 (1.40-1.80) |
| €20-39,999 | 88 | 3,022 | 2.91 | 1.54 (1.36-1.73) |
| <€20,000 | 225 | 8,704 | 2.59 | 1.52 (1.38-1.66) |
| **Mediterranean ((North African + Turkish) and non-Dutch native** | | |  |  |
| €80-100,00 | 84 | 4,917 | 1.71 | 1.43 (1.00-2.06) |
| €60-79,999 | 50 | 3,207 | 1.56 | 1.26 (0.88-1.79) |
| €40-59,999 | 43 | 3,173 | 1.36 | 2.01 (1.53-2.64) |
| €20-39,999 | 51 | 3,504 | 1.46 | 2.29 (1.79-2.92) |
| <€20,000 | 148 | 8,139 | 1.82 | 2.05 (1.67-2.51) |
| **Other African and non-Dutch native** | | |  |  |
| €80-100,00 | 87 | 4,047 | 2.15 | 1.16 (0.73-1.85) |
| €60-79,999 | 53 | 3,290 | 1.61 | 1.97 (1.43-2.70) |
| €40-59,999 | 57 | 2,083 | 2.74 | 1.60 (1.17-2.18) |
| €20-39,999 | 35 | 1,191 | 2.94 | 2.06 (1.66-2.55) |
| <€20,000 | 25 | 1,103 | 2.27 | 1.86 (1.62-2.14) |

**Table S20 (Continued)**

| **Household Disposable Income Quintile (euros)** | **SMM N** | **Total N** | **Rate %** | **aRR (95% CI)** |
| --- | --- | --- | --- | --- |
| **Other Asian and non-Dutch native** | | |  |  |
| €80-100,00 | 31 | 1,378 | 2.25 | 1.18 (0.95-1.47) |
| €60-79,999 | 38 | 1,085 | 3.50 | 1.09 (0.82-1.44) |
| €40-59,999 | 24 | 1,075 | 2.23 | 0.95 (0.70-1.29) |
| €20-39,999 | 28 | 1,084 | 2.58 | 1.03 (0.78-1.36) |
| <€20,000 | 61 | 2,025 | 3.01 | 1.30 (1.10-1.54) |
| **Missing ethnicity and Dutch native** | | |  |  |
| €80-100,00 | 34 | 2,313 | 1.47 | 1.55 (1.25-1.92) |
| €60-79,999 | 34 | 1,873 | 1.82 | 1.18 (0.90-1.55) |
| €40-59,999 | 25 | 1,286 | 1.94 | 2.03 (1.56-2.64) |
| €20-39,999 | 18 | 964 | 1.87 | 2.15 (1.54-3.00) |
| <€20,000 | 25 | 1,124 | 2.22 | 1.65 (1.11-2.45) |
| **Missing ethnicity and non-Dutch native** | | |  |  |
| €80-100,00 | 192 | 11,083 | 1.73 | 1.59 (1.12-2.27) |
| €60-79,999 | 229 | 10,703 | 2.14 | 2.52 (1.83-3.48) |
| €40-59,999 | 273 | 12,130 | 2.25 | 1.61 (1.08-2.41) |
| €20-39,999 | 316 | 14,539 | 2.17 | 1.85 (1.28-2.69) |
| <€20,000 | 593 | 27,817 | 2.13 | 2.20 (1.70-2.83) |
| **All minoritised ethnicity and non-Dutch native** | | |  |  |
| €80-100,00 | 324 | 18,602 | 1.74 | 1.21(1.07-1.36) |
| €60-79,999 | 349 | 17,124 | 2.04 | 1.43(1.28-1.60) |
| €40-59,999 | 410 | 19,067 | 2.15 | 1.52(1.37-1.69) |
| €20-39,999 | 521 | 23,206 | 2.25 | 1.59(1.45-1.75) |
| <€20,000 | 1,062 | 48,139 | 2.21 | 1.58(1.46-1.70) |

**Table S21.** Number of severe maternal morbidity (SMM) cases, total in each quintile, rate of SMM and adjusted risk ratio (aRR) and their 95% confidence intervals (95% CI) of Unemployed women and SMM compared to Caucasian Employed women stratified by ethnic and migration status and adjusted for age, parity, year.

| **Employment** | **SMM N** | **Total N** | **Rate** | **aRR (95% CI)** |
| --- | --- | --- | --- | --- |
| **Caucasian and Dutch native** | | |  |  |
| Employed | 6,680 | 467,259 | 1.43 | 1 (ref) |
| Unemployed | 1,716 | 88,415 | 1.94 | 1.35 (1.28-1.42) |
| **Caucasian and non-Dutch native** | | |  |  |
| Employed | 996 | 72,023 | 1.38 | 0.95 (0.89-1.02) |
| Unemployed | 493 | 31,587 | 1.56 | 1.07 (0.98-1.18) |
| **Minoritised ethnicity and Dutch native** | | | |  |
| Employed | 110 | 6,779 | 1.62 | 1.12 (0.93-1.36) |
| Unemployed | 41 | 1,700 | 2.41 | 1.66 (1.22-2.25) |
| **Other including mixed and non-Dutch native** | | | |  |
| Employed | 829 | 43,403 | 1.91 | 1.32 (1.22-1.41) |
| Unemployed | 910 | 43,768 | 2.08 | 1.44 (1.34-1.54) |
| **Mediterranean ((North African + Turkish) and non-Dutch native** | | | |  |
| Employed | 138 | 6,694 | 2.06 | 1.49 (1.26-1.77) |
| Unemployed | 158 | 5,733 | 2.76 | 1.98 (1.69-2.33) |
| **Other African and non-Dutch native** | | | |  |
| Employed | 191 | 8,434 | 2.26 | 1.56 (1.35-1.80) |
| Unemployed | 254 | 9,900 | 2.57 | 1.79 (1.58-2.03) |
| **Other Asian and non-Dutch native** | | |  |  |
| Employed | 186 | 12,184 | 1.53 | 1.05 (0.91-1.22) |
| Unemployed | 241 | 14,225 | 1.69 | 1.17 (1.03-1.34) |
| **Missing ethnicity and Dutch native** | | | |  |
| Employed | 208 | 10,924 | 1.90 | 1.36 (1.19-1.57) |
| Unemployed | 66 | 2,134 | 3.09 | 2.19 (1.72-2.79) |
| **Missing ethnicity and non-Dutch native** | | | |  |
| Employed | 108 | 4,467 | 2.42 | 1.70 (1.41-2.06) |
| Unemployed | 88 | 3,237 | 2.72 | 1.92 (1.55-2.37) |
| **All minoritised ethnicity and non-Dutch native** | | |  |  |
| Employed | 1,344 | 70,715 | 1.9 | 1.31(1.24-1.39) |
| Unemployed | 1,563 | 73,626 | 2.12 | 1.47(1.39-1.56) |

**Table S22.** Number of severe maternal morbidity (SMM) cases, total in each quintile, rate of SMM and adjusted risk ratios (aRR) and their 95% confidence intervals (95% CI) for women in each Educational Attainment group and SMM, compared to the women with the Caucasian women with the highest educational attainment stratified by ethnicity and migration status and adjusted for age, parity and year.

| **Individual Educational Attainment** | **SMM N** | **Total N** | **Rate %** | **aRR (95% CI)** |
| --- | --- | --- | --- | --- |
| **White and Dutch native** | | |  |  |
| High | 3,037 | 223,295 | 1.36 | 1 (ref) |
| Medium | 3,398 | 194,186 | 1.75 | 1.32 (1.25-1.39) |
| Low | 999 | 46,839 | 2.13 | 1.59 (1.48-1.71) |
| **White and non-Dutch native** | | |  |  |
| High | 429 | 28,079 | 1.53 | 1.10 (1.00-1.22) |
| Medium | 437 | 26,814 | 1.63 | 1.21 (1.09-1.34) |
| Low | 250 | 15,083 | 1.66 | 1.22 (1.07-1.39) |
| **Minoritised ethnicity and Dutch native** | | | |  |
| High | 37 | 2,845 | 1.30 | 0.95 (0.69-1.32) |
| Medium | 64 | 3,229 | 1.98 | 1.48 (1.15-1.89) |
| Low | 27 | 1,168 | 2.31 | 1.73 (1.18-2.52) |
| **Other including mixed and non-Dutch native** | | | |  |
| High | 271 | 14,126 | 1.92 | 1.39 (1.23-1.58) |
| Medium | 575 | 24,926 | 2.31 | 1.71 (1.57-1.88) |
| Low | 427 | 18,660 | 2.29 | 1.67 (1.51-1.85) |
| **Mediterranean ((North African + Turkish) and non-Dutch native** | | | |  |
| High | 50 | 2,018 | 2.48 | 1.93 (1.46-2.56) |
| Medium | 119 | 4,527 | 2.63 | 2.08 (1.73-2.51) |
| Low | 56 | 2,217 | 2.53 | 1.91 (1.46-2.49) |
| **Other African and non-Dutch native** | | | |  |
| High | 46 | 2,081 | 2.21 | 1.60 (1.20-2.14) |
| Medium | 152 | 5,805 | 2.62 | 1.95 (1.66-2.30) |
| Low | 157 | 5,485 | 2.86 | 2.13 (1.81-2.51) |
| **Other Asian and non-Dutch native** | | |  |  |
| High | 82 | 5,595 | 1.47 | 1.06 (0.85-1.32) |
| Medium | 76 | 4,196 | 1.81 | 1.34 (1.07-1.69) |
| Low | 73 | 3,936 | 1.85 | 1.35 (1.07-1.71) |
| **Missing ethnicity and Dutch native** | | | |  |
| High | 101 | 5,476 | 1.84 | 1.40 (1.15-1.71) |
| Medium | 111 | 4,616 | 2.40 | 1.86 (1.54-2.25) |
| Low | 36 | 1,172 | 3.07 | 2.35 (1.69-3.27) |
| **Missing ethnicity and non-Dutch native** | | | |  |
| High | 31 | 1,559 | 1.99 | 1.47 (1.03-2.10) |
| Medium | 63 | 1,953 | 3.23 | 2.46 (1.92-3.16) |
| Low | 40 | 1,285 | 3.11 | 2.33 (1.70-3.19) |

**Table S22. (Continued)**

| **Individual Educational Attainment** | **SMM N** | **Total N** | **Rate %** | **aRR (95% CI)** |
| --- | --- | --- | --- | --- |
| **All minoritised ethnicity and non-Dutch native** |  |  |  |  |
| High | 449 | 23,820 | 1.88 | 1.37(1.24-1.52) |
| Medium | 922 | 39,454 | 2.34 | 1.75(1.62-1.88) |
| Low | 713 | 30,298 | 2.35 | 1.73(1.59-1.88) |

**Table S23.** Adjusted risk ratios (aRR) and their 95% confidence intervals (95% CI) of Model 1 (adjusted for age, ethnicity, migration status, year and parity) where SES-WOA Welfare has been re-categorized to be the same size in each group as household income and N (%) of severe maternal morbidity (SMM) in each of the new groups.

| **SES-WOA Welfare** | **SMM N** | **Total N** | **Rate %** | **aRR (95% CI)** |
| --- | --- | --- | --- | --- |
| Most disadvantaged 20% | 2,351 | 117,443 | 2.00 | 1.25 (1.17-1.33) |
| More disadvantaged 20-40% | 1,840 | 108,932 | 1.69 | 1.09 (1.02-1.16) |
| Less disadvantaged 40-60% | 2,431 | 151,747 | 1.60 | 1.07 (1.02-1.14) |
| Less disadvantaged 20-40% | 3,131 | 206,046 | 1.52 | 1.04 (0.99-1.09) |
| Least disadvantaged 20% | 3,660 | 248,698 | 1.47 | 1 (ref) |
| ICC |  |  |  | 0.014(0.010-0.018) |

**Table S24.** Adjusted risk ratios (aRR) and their 95% confidence intervlas (95% CI) of Model 1(adjusted for age, ethnicity, migration status, year and parity) where SES-WOA Employment has been re-categorized to be the same size in each group individual employment status and N (%) of severe maternal morbidity (SMM) in each of the new groups.

| **SES-WOA Employment** | **SMM N** | **Total N** | **Rate %** | **aRR (95% CI)** |
| --- | --- | --- | --- | --- |
| Low | 3,772 | 200,065 | 1.89 | 1.14 (1.09-1.19) |
| High | 9,641 | 632,801 | 1.52 | 1.00 (ref) |
| ICC |  |  |  | 0.013 (0.010-0.018) |

**Table S25.** Adjusted risk ratio (aRR) and their 95% confidence intervals (95% CI) of Model 1 (adjusted for age, ethnicity, migration status, year and parity) where SES-WOA Education has been re-categorized to be the same size in each group individual educational attainment and N(%) of severe maternal morbidity (SMM) in each of the new groups.

| **SES-WOA Education** | **SMM N** | **Total N** | **Rate %** | **aRR (95% CI)** |
| --- | --- | --- | --- | --- |
| Low | 2,462 | 126,663 | 1.94 | 1.22 (1.15-1.28) |
| Medium | 5,506 | 343,138 | 1.60 | 1.08 (1.03-1.12) |
| High | 5,292 | 354,988 | 1.49 | 1.00 (ref) |
| ICC |  |  |  | 0.013(0.010-0.018) |

**Table S26.** Characteristics of women with and without missing data for Household Disposable Income, Individual Educational Attainment, and Liveability N(%).

|  | **Household Disposable Income** | | **Individual Educational Attainment** | | **Liveability** | |
| --- | --- | --- | --- | --- | --- | --- |
|  | **Not missing** | **Missing** | **Not missing** | **Missing** | **Not missing** | **Missing** |
|  | **740,948(89.0)** | **91,918(11.0)** | **651,171(78.2)** | **181,695(21.8)** | **738,887(88.7)** | **93,979(11.3)** |
| **SMM** |  |  |  |  |  |  |
| No | 728407 (98.3) | 91046 (99.0) | 640027 (98.3) | 179426 (98.8) | 727088 (98.4) | 92365 (98.3) |
| Yes | 12541 (1.7) | 872 (1.0) | 11144 (1.7) | 2269 (1.3) | 11799 (1.6) | 1614 (1.7) |
| **Parity** |  |  |  |  |  |  |
|  |  |  |  |  |  |  |
| Primiparous | 361649 (50.8) | 46407 (49.5) | 327717 (44.6) | 80339 (49.5) | 361744 (49.3) | 46312 (49.5) |
| Multiparous | 371701 (49.2) | 44996 (50.5) | 316689 (55.4) | 100008 (50.5) | 369082 (50.7) | 47615 (50.5) |
| **Age group** |  |  |  |  |  |  |
| <20 | 6763 (0.9) | 1080 (1.2) | 6561 (1.0) | 1282 (0.7) | 7006 (1.0) | 837 (0.9) |
| 20-25 | 61174 (8.3) | 8944 (9.7) | 56803 (8.7) | 13315 (7.3) | 62276 (8.4) | 7842 (8.3) |
| 25-30 | 220849 (29.8) | 27723 (30.2) | 205238 (31.5) | 43334 (23.9) | 220110 (29.8) | 28462 (30.3) |
| 30-35 | 285368 (38.5) | 34100 (37.1) | 249820 (38.4) | 69648 (38.3) | 283497 (38.4) | 35971 (38.3) |
| 35-40 | 137891 (18.6) | 16529 (18.0) | 110881 (17.0) | 43539 (24.0) | 137179 (18.6) | 17241 (18.3) |
| >40 | 28835 (3.9) | 3531 (3.8) | 21812 (3.4) | 10554 (5.8) | 28746 (3.9) | 3620 (3.9) |
| **Ethnicity** |  |  |  |  |  |  |
| Caucasian | 588889 (81.5) | 70395 (78.6) | 534296 (84.1) | 124988 (70.6) | 583348 (81.0) | 75936 (82.8) |
| Mediterranean (North African + Turkish) | 11041 (1.5) | 1480 (1.7) | 8846 (1.4) | 3675 (2.1) | 11339 (1.6) | 1182 (1.3) |
| Other African | 16363 (2.3) | 2414 (2.7) | 13751 (2.2) | 5026 (2.8) | 16824 (2.3) | 1953 (2.1) |
| Asian | 33710 (4.7) | 4939 (5.5) | 21831 (3.4) | 16818 (9.5) | 34475 (4.8) | 4174 (4.6) |
| Other including Mixed | 72584 (10.1) | 10289 (11.5) | 56386 (8.9) | 26487 (15.0) | 74425 (10.3) | 8448 (9.2) |
| **Mode of birth onset** |  |  |  |  |  |  |
| Induction | 177752 (24.8) | 21939 (24.7) | 156779 (24.9) | 42912 (24.5) | 177275 (24.8) | 22416 (24.6) |
| Primary Caesarean Birth | 59574 (8.3) | 7503 (8.4) | 49817 (7.9) | 17260 (9.8) | 59347 (8.3) | 7730 (8.5) |
| Spontaneous Birth | 479446 (66.9) | 59406 (66.9) | 423651 (67.2) | 115201 (65.7) | 477787 (66.9) | 61065 (67.0) |

**Table S26. (Continued)**

|  | **Household Disposable Income** | | **Individual Educational Attainment** | | **Liveability** | |
| --- | --- | --- | --- | --- | --- | --- |
|  | **Not missing** | **Missing** | **Not missing** | **Missing** | **Not missing** | **Missing** |
| **Place of birth** |  |  |  |  |  |  |
| At home | 91,955 (12.5) | 10,681 (11.7) | 82,902 (12.8) | 19,734 (10.9) | 90,654 (12.3) | 11,982 (12.8) |
| Birth centre | 19,297 (2.6) | 2,458 (2.7) | 17,035 (2.6) | 4,720 (2.6) | 19,397 (2.6) | 2,358 (2.5) |
| Hospital (1st line) | 74,976 (10.2) | 9,120 (10.0) | 65,861 (10.2) | 18,235 (10.1) | 74,718 (10.2) | 9,378 (10.0) |
| Hospital (2nd line) | 550,277 (74.7) | 69,087 (75.6) | 481,530 (74.4) | 137,834 (76.4) | 549,627 (74.8) | 69,737 (74.6) |
| En route | 59 (0.0) | 14 (0.0) | 56 (0.0) | 17 (0.0) | 69 (0.0) | NA |
| **Mode of birth** |  |  |  |  |  |  |
| Unassisted vaginal birth | 516,153 (72.9) | 63,399 (72.3) | 456,617 (73.3) | 122,935 (71.0) | 514,323 (72.8) | 65,229 (72.7) |
| Assisted vaginal birth | 62,523 (8.8) | 7,936 (9.1) | 55,579 (8.9) | 14,880 (8.6) | 62,534 (8.9) | 7,925 (8.8) |
| Elective caesarean birth | 63,222 (8.9) | 7,949 (9.1) | 52,841 (8.5) | 18,330 (10.6) | 62,966 (8.9) | 8,205 (9.1) |
| Emergency caesarean birth | 66,227 (9.4) | 8,393 (9.6) | 57,742 (9.3) | 16,878 (9.8) | 66,246 (9.4) | 8,374 (9.3) |
| Termination of pregnancy | 222 (0.0) | 34 (0.0) | 202 (0.0) | 54 (0.0) | 223 (0.0) | 33 (0.0) |
| **Migration background** |  |  |  |  |  |  |
| Dutch native | 518,263 (70.0) | 58,948 (64.1) | 482,826 (74.2) | 94,385 (52.0) | 510,236 (69.1) | 66,975 (71.3) |
| Moroccan | 26,592 (3.6) | 3,394 (3.7) | 22,464 (3.5) | 7,522 (4.1) | 26,934 (3.7) | 3,052 (3.3) |
| Other non-western | 61,637 (8.3) | 9,744 (10.6) | 39,800 (6.1) | 31,581 (17.4) | 63,821 (8.6) | 7,560 (8.0) |
| Other western | 81,217 (11.0) | 13,212 (14.4) | 57,962 (8.9) | 36,467 (20.1) | 83,943 (11.4) | 10,486 (11.2) |
| Surinamese | 16,661 (2.2) | 2,005 (2.2) | 15,751 (2.4) | 2,915 (1.6) | 16,819 (2.3) | 1,847 (2.0) |
| Turkish | 26,247 (3.5) | 3,297 (3.6) | 22,259 (3.4) | 7,285 (4.0) | 26,774 (3.6) | 2,770 (3.0) |
| Netherlands Antilles | 10,331 (1.4) | 1,318 (1.4) | 10,109 (1.6) | 1,540 (0.9) | 10,360 (1.4) | 1,289 (1.4) |
| **Dutch native** |  |  |  |  |  |  |
| Yes | 518,263 (70.0) | 58,948 (64.1) | 482,826 (74.2) | 94,385 (52.0) | 510,236 (69.1) | 66,975 (71.3) |
| No | 222,685 (30.0) | 32,970 (35.9) | 168,345 (25.8) | 87,310 (48.1) | 228,651 (31.0) | 27,004 (28.7) |

**Table S26. (Continued)**

|  | **Household Disposable Income** | | **Individual Educational Attainment** | | **Liveability** | |
| --- | --- | --- | --- | --- | --- | --- |
|  | **Not missing** | **Missing** | **Not missing** | **Missing** | **Not missing** | **Missing** |
| **Year** |  |  |  |  |  |  |
| 2013 | 91,122 (12.3) | 10,873 (11.8) | 76,288 (11.7) | 25,707 (14.2) | 90,733 (12.3) | 11,262 (12.0) |
| 2014 | 86,147 (11.6) | 10,540 (11.5) | 73,717 (11.3) | 22,970 (12.6) | 86,093 (11.7) | 10,594 (11.3) |
| 2015 | 77,398 (10.5) | 9,627 (10.5) | 67,209 (10.3) | 19,816 (10.9) | 77,288 (10.5) | 9,737 (10.4) |
| 2016 | 76,240 (10.3) | 9,663 (10.5) | 67,357 (10.3) | 18,546 (10.2) | 76,233 (10.3) | 9,670 (10.3) |
| 2017 | 74,675 (10.1) | 9,689 (10.5) | 66,179 (10.2) | 18,185 (10.0) | 74,694 (10.1) | 9,670 (10.3) |
| 2018 | 74,667 (10.1) | 9,391 (10.2) | 66,360 (10.2) | 17,698 (9.7) | 74,364 (10.1) | 9,694 (10.3) |
| 2019 | 81,207 (11.0) | 10,319 (11.2) | 72,592 (11.2) | 18,934 (10.4) | 81,203 (11.0) | 10,323 (11.0) |
| 2020 | 88,779 (12.0) | 10,813 (11.8) | 79,665 (12.2) | 19,927 (11.0) | 88,278 (12.0) | 11,314 (12.0) |
| 2021 | 90,713 (12.2) | 11,003 (12.0) | 81,804 (12.6) | 19,912 (11.0) | 90,001 (12.2) | 11,715 (12.5) |
| **Pre-existing medical conditions** |  |  |  |  |  |  |
| No | 526,509 (71.1) | 67,676 (73.6) | 457,442 (70.2) | 136,743 (75.3) | 527,440 (71.4) | 66,745 (71.0) |
| Yes | 214,439 (28.9) | 24,242 (26.4) | 193,729 (29.8) | 44,952 (24.7) | 211,447 (28.6) | 27,234 (29.0) |
| **Substance misuse or smoking** |  |  |  |  |  |  |
| No | 722,075 (97.4) | 89,813 (97.7) | 633,902 (97.4) | 177,986 (98.0) | 720,295 (97.5) | 91,593 (97.5) |
| Yes | 18,873 (2.6) | 2,105 (2.3) | 17,269 (2.6) | 3,709 (2.0) | 18,592 (2.5) | 2,386 (2.5) |
| **Mental health problems** |  |  |  |  |  |  |
| No | 656,956 (88.7) | 82,396 (89.6) | 575,349 (88.4) | 164,003 (90.3) | 656,164 (88.8) | 83,188 (88.5) |
| Yes | 83,992 (11.3) | 9,522 (10.4) | 75,822 (11.6) | 17,692 (9.7) | 82,723 (11.2) | 10,791 (11.5) |
| **Obesity** |  |  |  |  |  |  |
| No | 716,599 (96.7) | 89,230 (97.1) | 629,117 (96.6) | 176,712 (97.3) | 714,912 (96.8) | 90,917 (96.7) |
| Yes | 24,349 (3.3) | 2,688 (2.9) | 22,054 (3.4) | 4,983 (2.7) | 23,975 (3.2) | 3,062 (3.3) |

**Table S27.** Risk ratios and their 95% confidence intervals (95% CI) of the association between Household Disposable Income quintiles and severe maternal morbidity compared to the highest Household Disposable Income quintile using multiple imputation for missing data. Model 1* adjusted for age, ethnicity, migration status, year and parity. Model 2** additionally adjusted for pre-existing medical conditions, substance misuse, pre-existing mental health conditions. FMI= Fraction of missing information

| **Household Disposable Income Quintile** | **Univariable** | **Model 1*** | **Model 2**** |
| --- | --- | --- | --- |
| <€20,000 | 1.39 (1.32–1.47) | 1.34 (1.26–1.43) | 1.27 (1.20–1.35) |
| €20-39,999 | 1.30 (1.23–1.38) | 1.30 (1.22–1.38) | 1.23 (1.16–1.31) |
| €40-59,999 | 1.16 (1.10–1.22) | 1.19 (1.12–1.26) | 1.15 (1.08–1.21) |
| €60-79,999 | 1.05 (1.00–1.10) | 1.07 (1.02–1.13) | 1.05 (1.00–1.11) |
| €80-100,00 | 1 (ref) | 1 (ref) | 1 (ref) |
| FMI | 0.0635 | 0.0658 | 0.060 |

**Table S28.** Risk ratios and their 95% confidence intervals (95% CI) of the association between Individual Educational Attainment level and maternal morbidity compared to the highest Educational Attainment level using multiple imputation for missing data. Model 1* adjusted for age, ethnicity, migration status, year and parity. Model 2** additionally adjusted for pre-existing medical conditions, substance misuse, pre-existing mental health conditions. FMI= Fraction of missing information

| **Individual Educational Attainment** | **Univariable** | **Model 1*** | **Model 2**** |
| --- | --- | --- | --- |
| Low | 1.47 (1.40–1.55) | 1.41 (1.33–1.50) | 1.30 (1.29–1.39) |
| Medium | 1.28 (1.23–1.32) | 1.28 (1.23–1.34) | 1.21 (1.15–1.26) |
| High | 1 (ref) | 1 (ref) | 1 (ref) |
| FMI | 0.1485 | 0.1741 | 0.1755 |

References

1. Perined. *Perinatale zorg in Nederland anno 2020: landelijke perinatale cijfers en duiding. [Perinatal care in the Netherlands in 2020: national perinatal data and interpretation.]; [in Dutch]*. 2021; Available from: <https://assets.perined.nl/docs/3d6a2b46-aa8a-417e-a55e-de0184fe2078.pdf>.

2. CBS, *Documentatierapport GBA_Uniciteit-LMR 1995-2011 [Documentation report GBA_Unicity_LMR 1995-2011.] [in Dutch]*. 2013.

3. CBS, *Documentatie Ziekenhuisopnamen Landelijke Basisregstratie Ziekenhuiszorg (LBZBASISTAB) [Documentation Hospital admissions national registry hospitalcare]. [in Dutch]*. 2024.

4. Bakker, B.F.M., J. van Rooijen, and L. van Toor, *The System of social statistical datasets of Statistics Netherlands: An integral approach to the production of register-based social statistics.* Statistical Journal of the IAOS, 2014. **30**: p. 411-424.

5. buurt., C.B.v.d.S.S.-W.s.p.w.e.

6. Ministry of the Interior and Kingdom relations, n.d., *FAQ*.

7. <https://woononderzoek.nl/>, W.N.W.N.W.I.T.H.G.o.t.N.c.M.A.f.

8. D’Arcy, R.S., *Investigating the Health and Care Needs of Pregnant Women with Multiple Long-Term Conditions*. 2024.

9. Lee, S.I., et al., *Epidemiology of pre-existing multimorbidity in pregnant women in the UK in 2018: a population-based cross-sectional study.* BMC pregnancy and childbirth, 2022. **22**(1): p. 120.

10. Harron, K., et al., *Associations between pre-pregnancy psychosocial risk factors and infant outcomes: a population-based cohort study in England.* The Lancet Public Health, 2021. **6**(2): p. e97-e105.

11. Heslin, K.C., A. Elixhauser, and C.A. Steiner, *Hospitalizations involving mental and substance use disorders among adults, 2012.* 2015.

12. World Health Organization. Division of Mental, H., *The ICD-10 classification of mental and behavioural disorders : conversion tables between ICD-8, ICD-9 and ICD-10*. 1994, World Health Organization: Geneva.
